# *LbNR*-Derived Nitric Oxide Delays Lycium Fruit Coloration by Transcriptionally Modifying Flavonoid Biosynthetic Pathway

**DOI:** 10.3389/fpls.2020.01215

**Published:** 2020-08-13

**Authors:** Gen Li, Beibei Qin, Shuodan Li, Yue Yin, Jianhua Zhao, Wei An, Youlong Cao, Zixin Mu

**Affiliations:** ^1^ College of Life Sciences, Northwest A&F University, Yangling, China; ^2^ College of Grassland Agriculture, Northwest A&F University, Yangling, China; ^3^ National Wolfberry Engineering Research Center, Ningxia Academy of Agriculture and Forestry Sciences, Yinchuan, China

**Keywords:** Lycium, fruit ripening, nitric oxide (NO), nitric reductase (NR), anthocyanins, proanthocyanidins

## Abstract

Anthocyanin-derived fleshy fruit pigmentation has become an excellent system for studying the regulatory network underlying fruit ripening and quality. The transcriptional control of anthocyanin biosynthesis by MYB–bHLH–WDR complexes has been well established, but the intermediate signals through which the environmental or developmental cues regulate these transcription factors remain poorly understood. Here we found that nitric oxide (NO) production during Lycium fruit ripening decreased progressively presenting a negative relationship with anthocyanins. After cloning of the nitric reductase (*NR*) gene from *Lycium* *barbarum* (*LbNR*) plants, we demonstrated that *LbNR*-derived NO partially inhibited anthocyanin biosynthesis but enhanced proanthocyanidin (PA) accumulation, and delayed fruit coloration. Application of the NO donor, sodium nitroprusside (SNP), produced a similar effect. The endogenous or exogenous NO downregulated the transcripts both of the regulatory genes and the structural genes that related to anthocyanin biosynthesis, while upregulated both of those genes that related to PA biosynthesis. Given there is a significant negative relationship between the levels of anthocyanins and PAs during Lycium fruit ripening, NO not only inhibited anthocyanin *de novo* biosynthesis but redirected the flavonoid biosynthetic pathway from anthocyanins to PA production. Two types of LrMYB transcription factors of opposite nature, namely anthocyanin-specific and PA-specific, which belong to the R2R3-MYB subfamily and 1R-MYB subfamily, respectively, were identified from *L. ruthenicum* fruits. It was further found that NO acts by antagonizing the ABA signaling, a phytohormone we have previously shown playing a positive role in Lycium fruit coloration. Our results provided particularly novel information about NO–ABA–anthocyanin interplay during Lycium fruit development and ripening, which may fill a gap between the developmental cues and the transcriptional regulation of anthocyanin biosynthesis.

## Introduction

Nitric oxide (NO) was first identified as a unique, diffusible molecular messenger in animals, after which it emerged that NO in plants affects similar signal transduction pathways. Like that in mammalian systems, endogenous NO generation in plants occurs due to the catalytic action of two main enzymes, namely, NO synthase (NOS)-like and nitrate reductase (NR) (Chamizo-Ampudia et al., 2017; Del Castello et al., 2019; Kolbert et al., 2019). In general, the NOS family catalyzes the NADPH-dependent formation of NO, but there are no clear NOS homologs in plant genomes and no plant enzyme displaying NOS-like activity have been identified so far. Using nitrite as a substrate, plants generate NO by enzymatic and nonenzymatic routes. The former is mainly catalyzed by NR, a key enzyme involved in nitrogen assimilation (Chamizo-Ampudia et al., 2017; Del Castello et al., 2019). NR-derived NO is involved in many plant physiological processes ranging from growth regulation to the biotic and abiotic stress response.

Edible fleshy fruits, which provide a wide range of essential nutrients such as vitamins, antioxidants, and minerals, are natural and healthy foods (Gapper et al., 2013; Seymour et al., 2013). Fruit ripening is an important developmental event for fresh fruit-bearing plants because it determines the adaptation to the environment and the continuation of the species (Gapper et al., 2013; Seymour et al., 2013). Ripening time is also a very important agricultural trait for fleshy fruits because it satisfies people’s demand for fruits in different seasons. Therefore, fruit ripening regulation is a hot research topic for horticulturists and fruit tree experts. The available data have established the role of NO in senescence delay, in which NO acts primarily but not solely by limiting ethylene emission, resulting in delayed ripening (Manjunatha et al., 2010; Manjunatha et al., 2012; Bodanapu et al., 2016; Yang et al., 2016; Palma et al., 2019). NO application can also improve fruit quality, cold resistance, secondary metabolite biosynthesis, shelf life, and disease resistance (Li et al., 2017; Corpas et al., 2018; Corpas and Palma, 2018; Palma et al., 2019). Moreover, factors that induce NO generation can suppress fruit softening (Guo et al., 2018). Although experiments focused on unveiling the involvement of NO in fruit ripening have provided some insights into the biochemical events of this process, its comprehensive genetic regulation is yet to be understood (Manjunatha et al., 2010).

Anthocyanidins and anthocyanins are colored pigments in food and pharmaceutical ingredients with potential health benefits (Khoo et al., 2017). Their antioxidant activity has been extensively explored in a great number of plant species as well as in different organs from the same plant (Giampieri et al., 2018). Fleshy fruits are one of the main natural sources of this type of pigments. Anthocyanins accumulate during fruit ripening, so an increase in anthocyanin levels is a sign of fruit maturation. Anthocyanins significantly contribute to the quality characteristics of fruits and are therefore the targets of many breeding programs. The modification of anthocyanin metabolism by the genetic engineering of fruit maturation has been reported to enhance quality and shelf life (Jaakola, 2013; Zhang et al., 2013; Zhang et al., 2014). The anthocyanin composition in ripe fruit is established *via* complicated metabolic networks regulated by genetic, developmental, and environmental factors (Jaakola, 2013). Currently, the anthocyanin biosynthetic pathway is well known, and the key regulatory genes in many species that control the pathway have been identified, but the intermediate signals by which the environmental or developmental cues regulate anthocyanin biosynthesis remain poorly understood (Jaakola, 2013; Xu et al., 2015). It is reported in animal cells that plant-sourced flavonoid compounds make their function through the NO–ROS or NO–cGMP pathway (Gao et al., 2014; Qian et al., 2017; Zhang et al., 2018). Given that both anthocyanins and NO are involved in the regulation of fruit ripening, quality, and shelf life (Manjunatha et al., 2010; Zhang et al., 2013), whether there is a direct link between the NO signaling and anthocyanin metabolism remains unclear (Wang et al., 2018).

Wolfberry or goji (fruits from *Lycium barbarum* L. and *L.* *chinense* Mill) have been used in China as food and medicine for millennia, and globally, they are increasingly consumed as health foods (Potterat, 2010; Olatunji et al., 2016; Yao et al., 2018). Goji was usually consumed in the form of its dry fruit; however, with the improvement of people’s living conditions and increased health awareness, the market demand for fresh Lycium fruit is strongly increasing. Moreover, as with other bulk vegetables and fruits, the planting of Lycium indoors will be a trend in the future. These facts make the regulation of wolfberry ripening time and improvements in fresh fruit quality and shelf life a priority. However, basic research on the mechanisms behind the regulation of Lycium fruit ripening is very scarce. We recently demonstrated that phytohormone ABA enhanced Lycium fruit coloration/ripening by promoting anthocyanin biosynthesis (Li G. et al., 2019), at both genetic and pharmacological levels. In contrast, in the present work, we further found that *LbNR*-derived NO inhibited Lycium fruit coloration by suppressing anthocyanin *de novo* biosynthesis as well as by redirecting the flavonoid biosynthetic pathway from anthocyanin to proanthocyanidin (PA) production. Our results uncover a novel mechanism underlying developmental cues-mediated pigmentation regulation and suggest the possibility of engineering endogenous NO to control Lycium fruit ripening.

## Materials and Methods

### Plant Materials

Lycium fruits with two distinct colors [black fruit (*L*. *ruthenicum* Murr), BF and yellow fruit (*Lycium barbarum* L. var. *auranticarpum*), YF] were collected from 5-year-old trees at the Wolfberry (Lycium) Germplasm Repository of Ningxia [Academy of Agriculture and Forestry Sciences, Ningxia Hui Autonomous Region, China (38°38′N, 106°09′E; altitude, 1,100 m)]. For analysis of fruit ripening process, fresh Lycium fruits were sampled at five ripening stages (S1–S5) in the natural state as described by Zhao et al. (2015) with some minor modifications. The ripening process was divided in detail into the young fruit stage [S1, 9–10 days after anthesis (DAA)]; the green fruit stage (S2, 15–16 DAA); the early color breaker stage (S3, 20–22 DAA), the late color breaker stage (S4, 27–30 DAA); and the ripened fruit stage (S5, 30–34 DAA). The fruits that came from at least three different trees for the same ripening stage of each species were collected, immediately frozen in liquid nitrogen, and stored at −80°C prior to total RNA extraction and physiological analysis.

### Exogenous SNP Treatment

To avoid strong transpiration, the experiment was performed before evening. The surface of the green healthy fruits (at the S2 stage, 15 DAA) was sprayed with 1 mM sodium nitroprusside (SNP, Sigma-Aldrich, as treatment) or ddH_2_O (including 0.05% Tween 20, as a control), by a sprayer with a volume of 200 ml. The reagent was diluted with ddH_2_O containing 0.05% Tween 20 (to increase adhesion on the blade surface). The whole tree or partial branches within one tree were sprayed according to the amount of fruits they bore. The reagent was sprayed on at least three trees with at least 200 healthy fruits per tree of each Lycium species. Our experimental design was based on a randomized complete block design (RCBD). Mature fruits (at the S5 stage) were sampled 15 days after spraying, immediately frozen in liquid nitrogen, and stored at −80°C until further analysis.

### Determination of NO Production

0.1 g sample powder was suspended in 1 ml NO extraction solution [50 mM Tris-HCl, pH 7.8, 0.2 mM EDTA, 0.2% (v/v) Triton X-100, 2% (w/v) PVP, 10% (v/v) glycerol, and 5 mM DTT]. The mixture was placed on ice and simultaneously shaken at 80 rpm for 30 min. After centrifugation at 14,000× g for 40 min at 4°C, 500 µl supernatant was utilized in the NO assay.

The NO release was determined according to the method of Vitecek et al. (2008) and Airaki et al. (2012) with some minor modifications. DAF FM-DA (D2321, Sigma) was added to a freshly prepared crude extract solution at a 5 µM final concentration. Then, the reaction mixtures were incubated at 37°C in the dark for 20 min, after which fluorescence was measured using a Hitachi F7000 spectrofluorimeter (Hitachi High-Technologies Corporation, Tokyo, Japan) at excitation and emission wavelengths of 485 nm and 515 nm, respectively. As control reaction mixtures, fruit samples were preincubated for 30 min with 1 mM sodium tungstate, a nitrate reductase inhibitor, before the fluorescent probe was added. The fluorescence produced (NO release) was expressed as arbitrary units per milligram of fresh weight (FW). All samples were analyzed in biological triplicate, in which each biological replicate had at least 30 fruits to be tested.

### Anthocyanins Measurements

The amounts of anthocyanins in Lycium fruit extracts were determined spectrophotometrically as described by Li G. et al. (2019). The anthocyanin concentration was calculated according to a standard curve for which cyanidin-3-glucoside (626B021, Solarbio, China) was used as a standard. All samples were analyzed in biological triplicate, in which each biological replicate had at least 30 fruits to be tested.

### Proanthocyanidins Determination

The proanthocyanidins (PAs) in Lycium fruits were extracted according to the method of Pang et al. (2008), with some minor modifications. Briefly, 0.1 g fruit sample was extracted using 1 ml of PA extract solution (75% acetone solution containing 1% glacial acetic acid) by vortexing and then sonicated at room temperature for 1 h. After centrifugation at 2,500 g for 10 min, the residues were reextracted twice as above. The pooled supernatants were then extracted three times with chloroform and three times with hexane. After the crude extract was lyophilized with a vacuum freeze dryer (LYOQUEST-85 plus) for 16 h, the dry powder was thoroughly dissolved in 1 ml of ultrapure water, and the liquid was then sterilized by passing through a 0.22 μm reinforced nylon membrane filter (Billerica, MA, USA).

PA level is determined by a method of High Performance Liquid Chromatography (HPLC) as described by Tian et al. (2017). For HPLC detection, an Agilent ZORBAX Eclipse Plus C18 column (4.6 × 100 mm, 1.8 μm) was used at the following chromatographic conditions: mobile phase A (water:formic acid:trifluoroacetic acid = 97.9:2:0.1): mobile phase B (acetonitrile:water:formic acid:trifluoroacetic acid = 48:49.9:2:0.1) = 8:2; a 280 nm detection wavelength; a 1.0 ml/min flow rate; a 10 μl injection volume; and a 24°C column temperature. The standard curve was drawn using the peak areas of different concentrations of (+)-catechin. The PA concentration was expressed as milligram (mg) PAs per gram (g) fresh weight. All samples were analyzed in biological triplicate, in which each biological replicate had at least 30 fruits to be tested.

### ABA Extraction and Assay

Endogenous ABA was extracted from Lycium fruits according to the method described by Li G. et al. (2019), and ABA content was assayed using a Phytodetek Immunoassay Kit (PDK 09347/0096, Agdia, USA) according to the manufacturer’s instructions. Briefly, 0.2 g of fruit sample was fully ground with 1 ml of precooled 80% methanol extract solution (containing 200 mg·L^−1^ 2,6-di-tert-butyl-p-cresol, and 500 mg·L^−1^ citric acid monohydrate). After overnight leaching at 4°C, the mixture was centrifuged at 10,000 rpm and 4°C for 15 min, and the above-described procedure was repeated with the supernatant. The twice-treated supernatant was then combined, and the ground fruit was concentrated and dried with a Visible Nitrogen Blower (KD200, ALLSHENG, China). Finally, 0.8 ml of precooled 80% methanol was added to the dry powder, and the mixture was mixed with a vortex shaker to form the crude extract of ABA. All samples were analyzed in biological triplicate, in which each biological replicate had at least 30 fruits to be tested.

### Cloning the Full-Length Sequence of *LbNR* cDNA

Leaves from *L. barbarum* (collected from 5-year-old trees at the Wolfberry Germplasm Repository of Ningxia, China) were used for total RNA isolation with the TRIzol method (Invitrogen, Carlsbad, CA, USA), and the first strand of cDNA was synthesized from 1 μg total RNA using the reverse transcriptase M-MLV (TaKaRa Biotechnology, Dalian, China) and oligo (dT) according to the manufacturer’s instructions. According to the EST sequence alignment of *L*. *barbarum*, the primers of the intermediate fragment homologous to the *NR* gene of *Solanaceae* were designed, and the fragment was amplified. For rapid amplification of cDNA 3′-end (3′-RACE), the gene-specific primers (GSP), 3′SPI and 3′GSPII were designed, and F1 (3′GSPI), R1 (3′outer), F2 (3′GSPII), and R2 (3′inner) were subjected to nested PCR amplification. The PCR product was ligated into a pMD18-T vector for sequencing to obtain a 3′-end fragment. Simultaneously, the cDNA was treated with RNase H (TaKaRa Biotechnology, Dalian, China), dCTP, and Terminal Deoxynucleotidyl Transferase, and the capped 5′-end of the cDNA was used as a template for 5′-RACE. The nested PCR and multiple PCR procedures were referenced to 3′-RACE, and the PCR product was ligated into a pMD18-T vector for sequencing, to obtain a 5′-end fragment. Finally, the entire cDNA coding region was combined, reamplified, inserted into the pMD18-T vector, and sequenced and was hereby designated as *LbNR* (NCBI accession no. MK169415). The sequences of the universal primers 3′outer, 3′inner, 5′outer, and 5′inner as well as the sequences of the gene-specific primers 5′GSPI, 5′GSPII, 3′GSPI and 3′GSPII are shown in the **Appendix** (**Table S1**).

### Bioinformatic Analysis of *LbNR*

The phylogenetic tree of *LbNR* was constructed by MrBayes software and further beautified by the web page of https://itol.embl.de. The protein secondary structure was predicted by the web page of http://pfam.xfam.org/search.

### Virus-Induced *LbNR* Gene Silencing

The CDS fragment of the *LbNR* gene carrying the adaptor sequence was obtained by a pair of amplification primers (5′- ATGGCTGCATCTGTTGAAAAT; 3′- AATTTATTACTGCAGATTGTTGTTA). The pTRV2 vector (Liu et al., 2002) was digested with PstI to not only linearize the vector, but also expose the adaptor sequence. Both the linearized vector and *LbNR* product were digested with T4 DNA polymerase to produce the sticky ends. The PCR product was ligated to the sticky end of the vector and then transformed into E. *coli* to screen for pTRV2-derivative colonies with the silenced target gene (pTRV2-*LbNR*). After pTRV1 and pTRV2 or pTRV2-*LbNR* was rapidly frozen by liquid nitrogen, they were transfected with *Agrobacterium tumefaciens* strain GV3101 by heat shock.

For infiltration, a single colony was selected, inoculated in 5 ml of Luria–Bertani medium containing appropriate antibiotics (50 mg L^−1^ rifampicin, 50 mg L^−1^ kanamycin), and grown overnight in a 28°C shaker. The next day, the agrobacterium cultures were harvested by centrifugation at 4,000× g for 15 min, and the cells were resuspended in the infiltration medium (10 mM MES, 10 mM MgCl_2_, and 200 μM acetosyringone). After adjusting OD_600_ to 1.0, the cells were incubated at room temperature for 3–4 h. Subsequently, the pTRV1 and pTRV2 (Control) or pTRV2-derivative solutions were mixed at a ratio of 1:1 for injection. The VIGS analysis was performed in the green fruit stage (S2) for both of the species. The injection method was as described by Li G. et al. (2019) using a 1 ml syringe, and the needle was directed at the fruit growth point for pressure injection. The injection was conducted on at least three trees with at least 200 healthy fruits per tree of each treatment, for each species. For the fruit coloration rate assay, the fruits were collected at an interval of 24 h after injection, while for other analyses, the fruits were sampled 3 days after injection. All the sampled fruits were immediately frozen in liquid nitrogen and stored at −80°C until further analysis.

### Virus-Induced *LbNCED1* Gene Silencing

*LbNCED1* (9-cis-epoxycarotenoid dioxygenase 1)-VIGS fruits were constructed as described by Li G. et al. (2019).

### Determination of the Fruit Coloration Rate

The changes of fruit anthocyanin level were determined at 0, 1, 2, and 3 days after injection. The fruit coloration rate was reported as milligram anthocyanin (mg) per gram fresh weight fruit (g^−1^FW) per day (d^−1^).

### Real-Time PCR

Total RNA was extracted from the fruit tissues using a RNAprep Pure Plant Kit (DP432, TianGen, Beijing, China), and the cDNA was synthesized through reverse transcription reaction by a PrimeScript RT reagent kit (Takara, Dalian, China). Genomic DNA was removed using a RNase Free DNase I kit (Takara, Dalian, China) as instructed by the manufacturer. For the relative gene expression analysis, the housekeeping gene *LbActin* (HQ415754) was implemented as an internal control as it exhibits a uniform expression pattern in Lycium plants (Zeng et al., 2014).

Quantitative PCR amplifications were conducted with the BioRad CFX96 Touch™ Real-Time PCR Detection System (USA) using the SYBR Premix Ex Taq (Takara, Dalian, China). Each 15 µl reaction contained 7.5 µl SYBR Premix Ex Taq mix, 1.0 µl cDNA template (containing 100 ng of cDNA), 1.2 µl primer mix (0.6 µl each of the forward and reverse primers), and 5.3 µl ddH_2_O. The amplification program was as follows: one cycle of 30 s at 95°C, followed by 39 cycles of 5 s at 95°C, 30 s at 60°C, and 30 s at 72°C. The data were analyzed by the 2^–ΔΔCt^ method. The primers used for RT-qPCR are described in **Table S2**.

### Statistical Analysis

Statistical analyses were performed using SPSS version 19.0. Parameter differences among varied fruits’ developmental stages or different treatments were determined using one-way ANOVA with appropriate *post hoc* analysis. The column figures were drawn by Origin 9.0.

## Result

### Cloning and Bioinformatic Analysis of NR Gene in Lycium Plants

**Figure 1:**
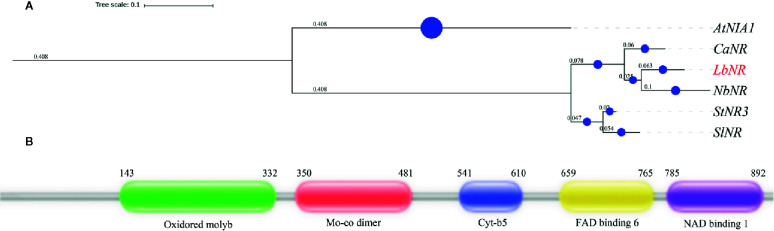
Bioinformatic analysis of the *LbNR* gene. The phylogenetic tree **(A)** of *LbNR* was constructed by MrBayes and further beautified by the https://itol.embl.de webpage. The protein secondary structure **(B)** was predicted by the http://pfam.xfam.org/search.

A *NR* gene in L*. barbarum* (*LbNR*) was successfully cloned by RACE technology. The gene has 3,193 bases, of which 145 bases are contained in the 5′ untranslated region (UTR), 312 bases are contained in the 3′ UTR, and 2,736 bases comprise the coding sequence (CDS). The *LbNR* CDS is translated to 911 amino acids. The cDNA sequence has been deposited in the NCBI nucleotide sequence database under accession number MK169415. A phylogenetic tree containing *LbNR* and some plants of the *Solanaceae* family (*Capsicum annuum*, *Solanum tuberosum*, *Nicotiana benthamiana*, *Solanum lycopersicum*) was constructed (**Figure 1A**). The tree was rerooted to the *Arabidopsis thaliana* *NR* gene *AtNIA1*. In the phylogenetic tree, the one with the highest homology to the gene *LbNR* is *NbNR* of *Nicotiana benthamiana* followed by *CaNR* of *Capsicum annuum*.

The amino acid sequence was used for secondary structure prediction (**Figure 1B**). The *LbNR* protein has five domains. Among them, the oxidoreductase molybdopterin binding domain (Oxidored molyb) and the Mo-co oxidoreductase dimerization domain (Mo-co dimer) bind to a molybdenum cofactor (MoCo). The cytochrome b5-like heme/steroid binding domain (Cyt-b5) binds to heme, and the oxidoreductase FAD-binding domain (FAD-binding-6) binds to FAD. When the FAD-binding domain receives electrons from NAD(P)H bound by the oxidoreductase NAD-binding domain (NAD-binding-1), the Cyt-b5 domain shuttles electrons to the Mo-co dimer, and the Mo-co dimer transfers the electrons to nitrate, completing the catalytic cycle.

### Nitric Oxide and Proanthocyanidin Changes During Lycium Fruit Ripening

**Figure 2:**
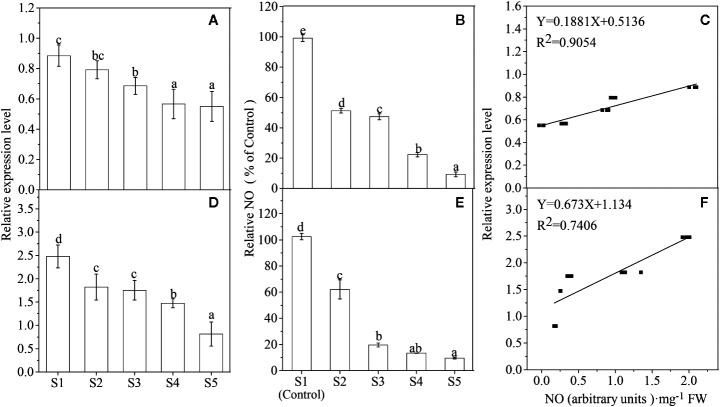
Changes of *LbNR* gene expression **(A, D)** and NO release **(B, E)** as well as their relationship **(C, F)** during Lycium fruit ripening (p < 0.05). S1–S5 represents the different developmental stages of Lycium fruits as described in the section of *Materials and Methods*. The fruit samples were collected at the same time from the same trees that bear fruits at all five developmental stages, and at least three trees of YF **(A–C)** and BF **(D–F)**, respectively, were selected. In **(B, E)**, the NO release was converted to relative values, in which the value of the S1 stage was taken as 100%. The error bars represent the SDs of three independent replicates. Different letters on the bars for the same species indicate significant differences between the treatments (p < 0.05).

To explore the role of NO in fruit maturation, both the NO release and the *LbNR* mRNA level were examined in two different colors of Lycium fruits, black fruits in *L*. *ruthenicum* Murr (BF) and yellow fruits in *Lycium barbarum* L. var. *auranticarpum* (YF), during their ripening process. It is shown that both NO production and *LbNR* transcript amount decreased gradually during Lycium fruits ripening, and there is a significant positive relationship between them for each species (**Figure 2**).

**Figure 3:**
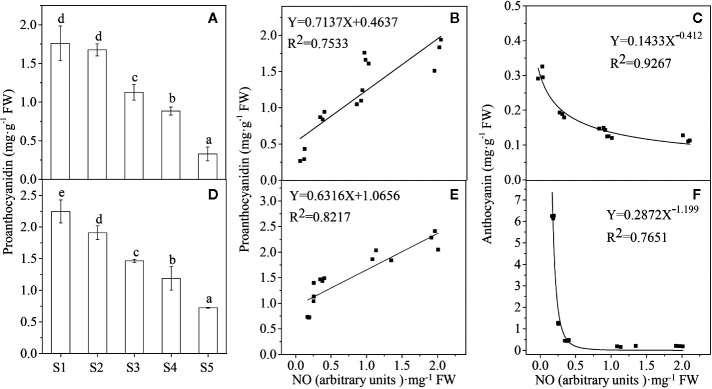
The relationship between the NO release and the pigment content. Proanthocyanidin (PA) concentration declines progressively during the process of Lycium fruit ripening **(A, D)**, and there is a significant positive relationship between PA and NO **(B, E)** while a negative relationship between anthocyanin and NO **(C, F)**, both for YF **(A–C)** and BF **(D, F)**. For **(B, F)**, p < 0.05, and for **(C, E)**, p < 0.01. The error bars represent the SDs of three independent replicates. Different letters on the bars for the same species indicate significant differences between the treatments (p < 0.05).

We have previously investigated anthocyanin levels during the different stages of ripening (S1–S5) in those fruits (Li G. et al., 2019). The present work found that in contrast to anthocyanins, the PA content decreased progressively (**Figure 3**) as these two colors of Lycium fruits ripened. There is a significant positive relationship between PAs and NO for each species while a negative relationship between anthocyanins and NO (**Figure 3**). Similarly, both the PA biosynthesis-related structural genes, *e.g*., *LbANR* and *LbLAR* (Chen et al., 2017), and the regulatory genes, *e.g.*, *LrMYB30* (Yan et al., 2017), a homolog of *AtTT2* (At5G35550), and *Lr* *TTG1-like* (Cluster-26021.71813) (Yan et al., 2019), a homolog of *AtTTG1* (AT5G24520.1) in *Arabidopsis*, were gradually downregulated during the ripening process (**Figure S1**).

### Effect of Virus-Induced *LbNR* Gene Silencing on Lycium Fruit Coloration

**Figure 4:**
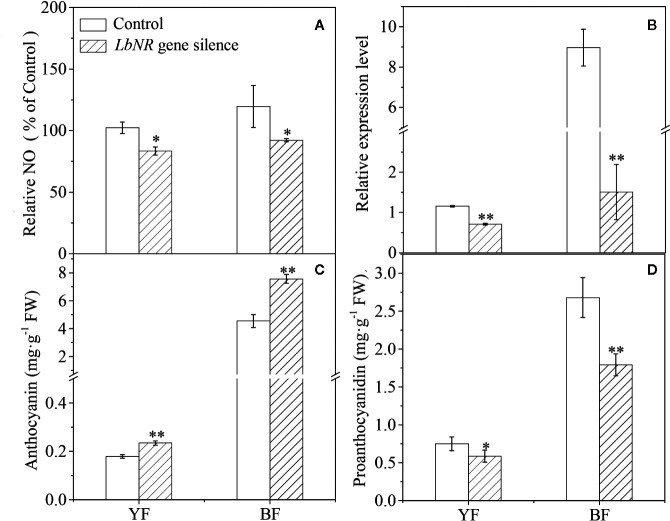
Effect of virus-induced *LbNR* gene silencing on anthocyanin and PA accumulation in Lycium fruits. pTRV-*LbNR* vector construction and fruit injection were as described in *Materials and Methods*. After 3 days of Agrobacterium injection, the healthy modified YF or BF were sampled, immediately frozen in liquid nitrogen, and stored at −80°C to assay the NO release by the DAF FM-DA-mediated spectrofluorometric method **(A)**, to detect *LbNR* transcript by qRT-PCR **(B)**, to document anthocyanin and PA content by the spectrophotometric method **(C)**, or by HPLC method **(D)**, respectively. In **(A)**, the NO release was converted to relative values, in which the value of the empty vector-injected fruits was taken as 100%. The error bars represent the SDs of three independent replicates. The asterisks on the bars for the same species indicate significant differences between the treatments. “*” indicates p < 0.05, and “**” indicates p < 0.01.

To further study the effect of NR-derived NO on fruit ripening, we constructed virus-induced gene silencing (VIGS)-*LbNR* fruits. Given that both the *LbNR* transcript abundance and NO release were significantly decreased in comparison with the control values (injected with empty vector), we successfully silenced the *LbNR* gene (**Figures 4A, B**). The anthocyanin content in *LbNR* gene-silenced fruits was higher than that in the control fruits. In contrast, *LbNR* silencing significantly decreased PA accumulation in mature Lycium fruits (**Figures 4C, D** **)**. It is further validated from the molecular genetic level that NO negatively correlated with anthocyanins, but positively with PAs in Lycium fruits.

**Figure 5:**
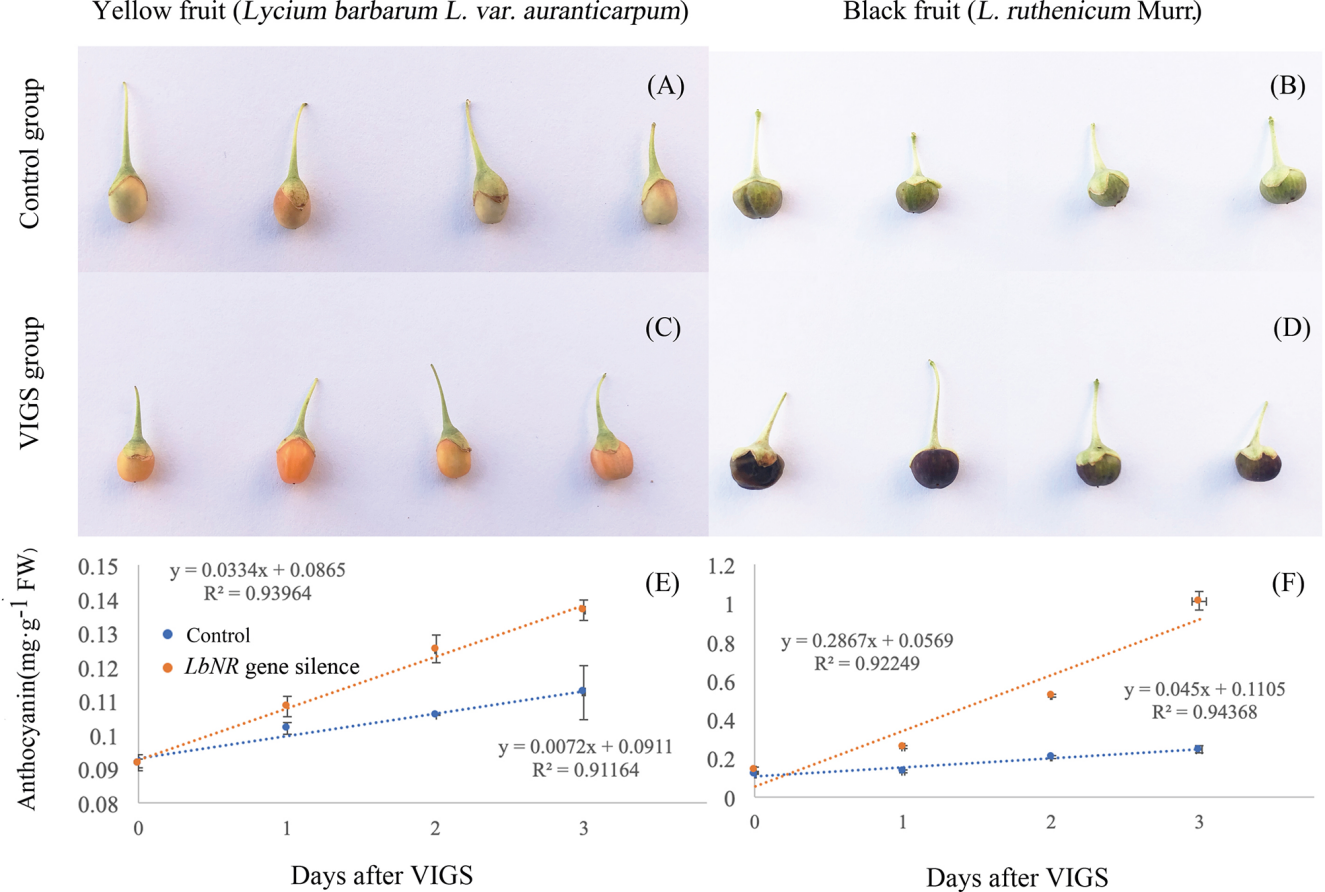
The fruit phenotypes and coloration rates after virus-induced *LbNR* gene silencing. **(A, B)** Phenotypes of YF and BF at 2 d after injection with the pTRV empty vector, respectively. **(C, D)** Phenotypes of YF and BF at 2 d after *LbNR* gene silencing, respectively. **(E, F)** The coloration rates within 3 d after VIGS injection of YF and BF, respectively. For injection, the needle is inserted into the growing point of the green stage fruits (S2) for pressure injection. The error bars represent the SDs of three independent replicates. The asterisks on the bars for the same species indicate significant differences between the treatments (p < 0.01).

To compare the fruit ripening process in the control group (**Figures 5A, B**) with that in the VIGS group (**Figures 5C, D**), the fruit coloration rate was also surveyed. It is shown that *LbNR* silencing significantly facilitated fruit coloration both for the yellow and black color Lycium fruits (**Figures 5E, F**).

### Effect of *LbNR*-Silencing on the Gene Expression of Flavonoid Biosynthetic Pathway

**Figure 6:**
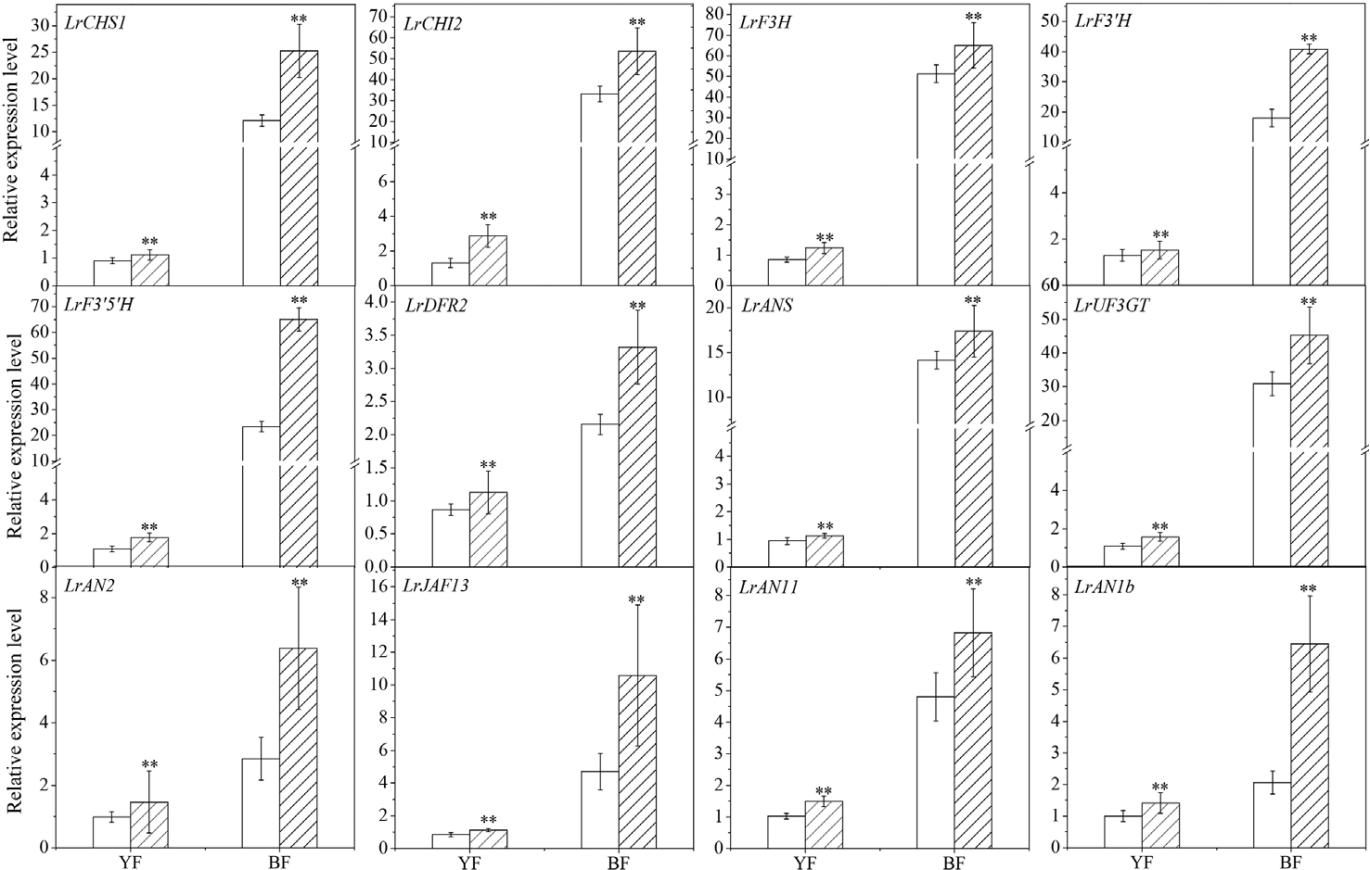
Effect of virus-induced *LbNR* gene silencing on anthocyanin biosynthesis-related gene expression in Lycium fruits. Transcript levels of *LrCHS1*, *LrCHI2*, *LrF3H*, *LrF3′H*, *LrF3′5 ′H*, *LrDFR2*, *LrANS*, and *LrUF3GT*, belonging to the structural genes, and transcript levels of *LrAN2*, *LrJAF13, LrAN11*, and *LrAN1b*, belonging to the MYB–bHLH–WD40 transcription factors, were shown. The gene expression differences between the two species were compared using YF (injected with empty vector) as the control for calculating the relative expression levels of the indicated genes. The data show the means ± SDs (n = 3). The asterisks on the bars for the same species indicate significant differences between the treatments. “**” indicates p < 0.01.

To explore the mechanism underlying the effect of NR-derived NO on anthocyanin biosynthesis during Lycium fruit ripening, the transcript abundance of genes in the flavonoid biosynthetic pathway was determined by qRT-PCR (**Figure 6**). *LbNR* silencing significantly upregulated the expression of several structural genes, *e.g.*, *LrCHS1* (chalcone synthase 1b, KC794742), *LrCHI2* (chalcone isomerase, KF031377), *LrF3H* (flavanone 3-hydroxylase, KC794744), *LrF3′H* (flavonoid 3-hydroxylase, KF732853), *LrF3′5 ′H* (flavonoid 3, 5-hydroxylase), *LrDFR* (dihydroflavonol-4-reductase-like, KF031379)*, LrANS* (anthocyanidin synthase, KC794745) and *LrUF3GT* (UDP glucose flavonoid 3-glucosyl transferase, KF768073). These genes have been isolated from *L.* *ruthenicum*, and their transcript amounts were positively correlated with anthocyanin content in this fruit, except *LrCHI* (Zeng et al., 2014). On the contrary, *LbNR* silencing significantly downregulated the structural genes involved in PA biosynthesis (**Figure S2**), *e.g.*, *LbANR* and *LbLAR* (Chen et al., 2017).

Similar to its effect on the structural genes, *LbNR* silencing also significantly upregulated the expression of the regulatory genes encoding MYB–bHLH–WD40 transcription factors (TFs) (**Figure 6**) specific to anthocyanin biosynthesis, including *LrAN2* (anthocyanin 2, KF768075) in the R2R3 MYB family, *LrJAF13* (KF768076) in the bHLH family, *LrAN11* (anthocyanin 11, KY131959) and *LrAN1b* (anthocyanin 1b, KF768077) in the WD40 family (Zeng et al., 2014). This effect is more obvious for BF than for YF because the former has a higher anthocyanin content. In addition to their genetic variation, a gene-specific sensitivity to endogenous NO was also observed in both the structural and the regulatory genes in Lycium fruits. On the contrary, *LbNR* silencing significantly downregulated genes encoding MYB–bHLH–WD40 TFs specific to PA biosynthesis (**Figure S2**), *e.g.*, *LrMYB30* and *LrTTG1-like*.

### Exogenous SNP Reduced Anthocyanin Biosynthesis but Enhanced PA Biosynthesis

**Figure 7:**
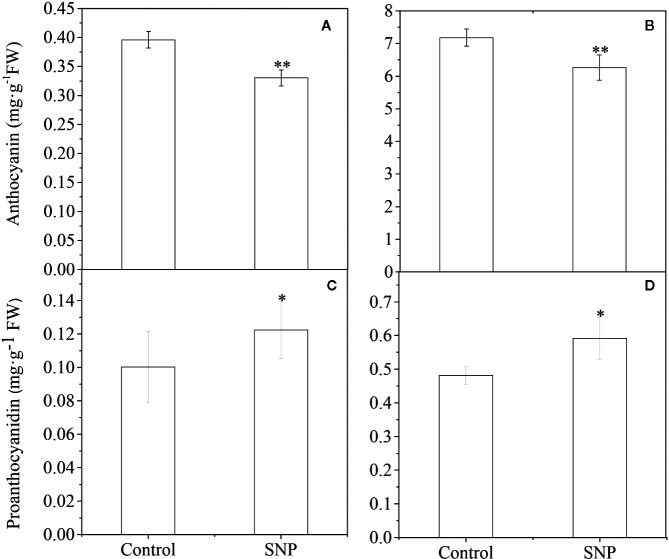
Effect of exogenous SNP application on anthocyanin and PA accumulation in Lycium fruits. 1 mM SNP (soluted in 0.05% Tween 20) was utilized to spray the green stage fruits (S2) of YF and BF, on a sunny day after the sun went down. Fruits were sprayed with only a 0.05% Tween 20 solution as a control. After 15 days, the mature fruits (S5) were sampled to measure anthocyanin **(A, B)** and PA **(C, D)** content for both of YF **(A, C)** and BF **(B, D)**, respectively. The data show the means ± SDs (n = 3). The asterisks on the bars for the same species indicate significant differences between the treatments. “*” indicates p < 0.05, and “**” indicates p < 0.01.

NO molecules released by a donor can either mimic an endogenous NO-related response or substitute for an endogenous NO deficiency (Del Castello et al., 2019; Kolbert et al., 2019). Therefore, we examined the effect of spraying sodium nitroprusside (SNP), one of the most common NO donors, on Lycium trees on anthocyanin accumulation. Exogenous SNP decreased anthocyanin levels in ripened black and yellow Lycium fruits (**Figures 7A, B**) but enhanced PA accumulation for both of the fruits compared to that observed in control fruits (**Figures 7C, D**).

**Figure 8:**
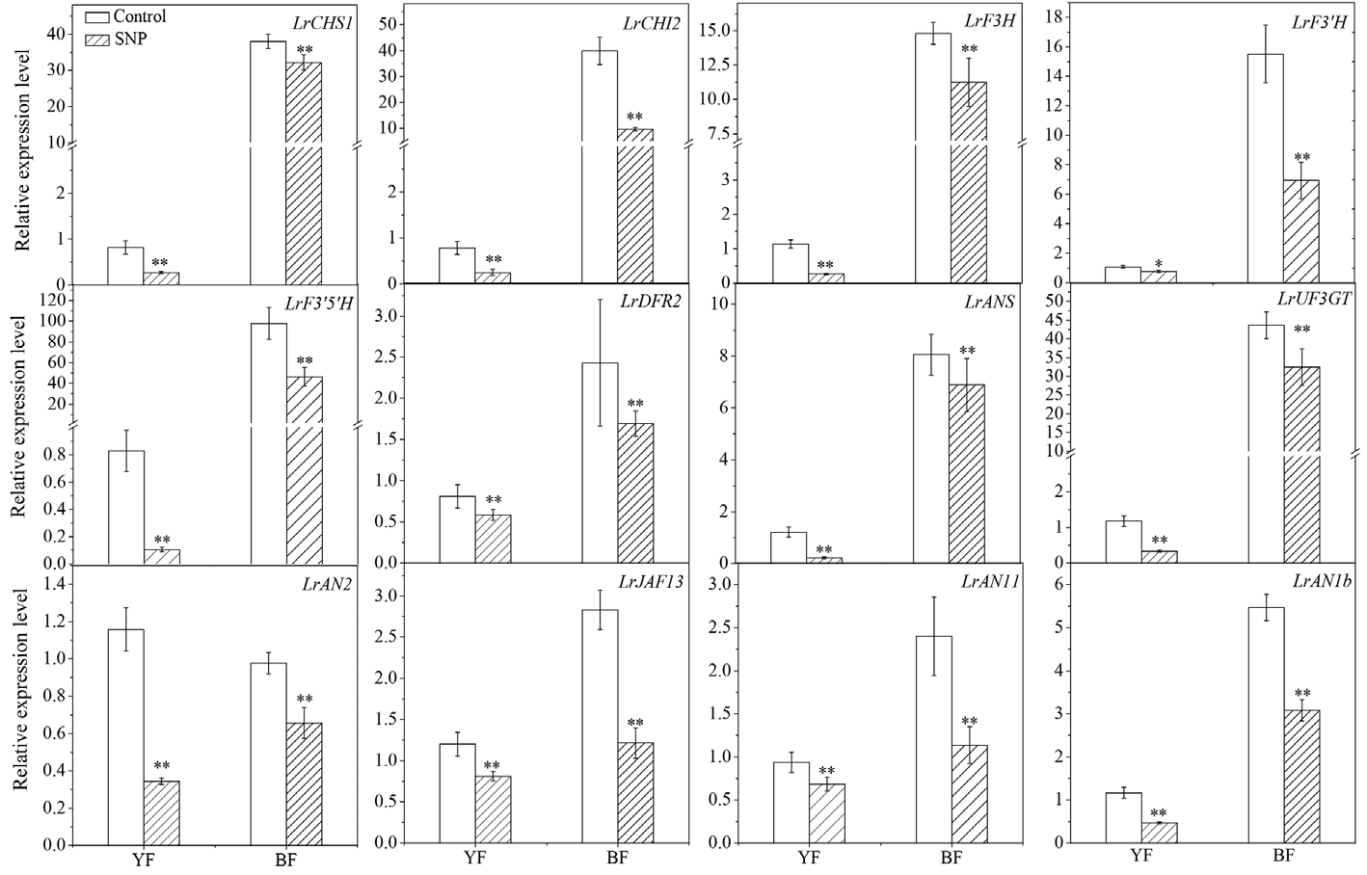
Effect of exogenous SNP application on anthocyanin biosynthesis-related gene expression in Lycium fruits. Transcript levels of *LrCHS1*, *LrCHI2*, *LrF3H*, *LrF3′H*, *LrF3′5 ′H*, *LrDFR2*, *LrANS*, *LrUF3GT LrAN2*, *LrJAF13*, *LrAN11*, and *LrAN1b* were shown. The gene expression differences between the two species were compared using YF (sprayed with ddH_2_O) as the control for calculating the relative expression levels of the indicated genes. The data show the means ± SDs (n = 3). The asterisks on the bars for the same species indicate significant differences between the treatments. “*” indicates p < 0.05, and “**” indicates p < 0.01.

SNP application significantly downregulated the transcription of both the structural genes *LrCHS1*, *LrCHI2*, *LrF3H*, *LrF3′H*, *LrF3′5 ′H*, *LrDFR2*, *LrANS*, and *LrUF3GT* and the regulator genes *LrAN2*, *LrJAF13, LrAN11*, and *LrAN1b* in the flavonoid biosynthetic pathway (**Figure 8**). The implementation of SNP significantly upregulated genes involved in PA biosynthesis, both for the structural genes, *e.g.*, *LbANR* and *LbLAR* (**Figures S3A, B**), and the regulatory genes, *e.g.*, *LrMYB30* and *Lr TTG1-like* (**Figures S3C, D**). This transcriptional response was observed in both colors of fruits following SNP application, although there was a genotype- and gene-specific pattern. Therefore, we concluded that exogenous SNP decreased the transcripts of genes related to anthocyanin biosynthesis but enhanced that of genes related with PA biosynthesis.

### Nitric Oxide Delays *L. ruthenicum* Fruit Coloration by Antagonizing ABA Action

**Figure 9:**
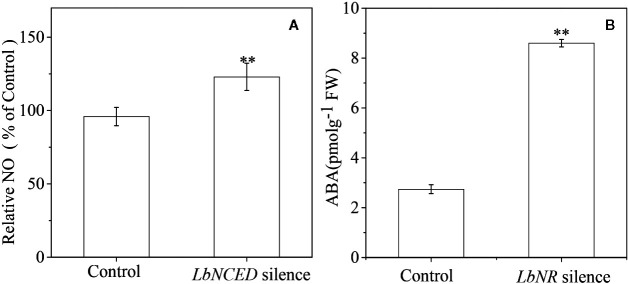
Effect of *LbNR* silencing or *LbNCED1* silencing on ABA accumulation and NO release of *L*. *ruthenicum* fruits, respectively. ABA was assayed by an immunoassay method as described in the section of “*Materials and Methods*”, and NO was determined as described in **Figure 2**. The error bars represent the SDs of three independent replicates. The asterisks on the bars for the same species indicate significant differences between the treatments (p < 0.01).

Our previous studies have shown that ABA acts as one of the main positive regulators of Lycium fruit coloration/ripening (Li G. et al., 2019) by activating anthocyanin biosynthesis. In the present work, we further determined the interaction between ABA signaling and NO signaling in BF coloration. It is shown that *LbNR* silencing significantly promotes the accumulation of ABA in BF, while *LbNCED1* silencing clearly enhances the release of NO (**Figures 9A, B**). There is a significant negative relationship between the release of NO and the accumulation of ABA as well as between the transcripts of *LbNR* and *LbNCED1* during fruit ripening (**Figure S4**). Unlike NO enhanced PA biosynthesis, ABA inhibited it (**Figure S5**). Moreover, except *LrAN2* and *LrMYB30*, we identified the other MYB TFs in BF that were activated by NO but inactivated by ABA, *vice versa*, e.x. *LrMYB1*, *LrMYB3*, *LrMYB30*, *LrMYB44*, and *LrMYB73* (**Figures S6** and **S7**). Among these MYB TFs, *LrAN2*, *LrMYB1*, *LrMYB3*, and *LrMYB44* belong to the R2R3-MYB subfamily, while *LrMYB30* and *LrMYB73* belong to the 1R-MYB subfamily (Yan et al., 2017). Our results indicated that NO and ABA antagonized each other to regulate the coloration of Lycium fruits.

## Discussion

Fruit ripening is a highly regulated developmental process involving drastic internal transcriptional and biochemical modifications that coincide with seed maturation (Corpas et al., 2018). As a consequence of its action as an important gaseous signaling molecule in prokaryotes and eukaryotes, NO regulates critical developmental transitions and stress responses in plants (Del Castello et al., 2019; Kolbert et al., 2019). NO promotes leaf expansion, represses floral transition, stimulates light-dependent germination, promotes de-etiolation, inhibits maturation, and senescence, (Bruand and Meilhoc, 2019). Like in pepper, tomato, strawberry, and avocado plants (Gonzalez-Gordo et al., 2019; Palma et al., 2019), the present work indicated that the release of NO progressively declined during fruit ripening in Lycium plants, a medicinal and edible species. These results may show that it is a conserved event for endogenous NO production decline during fresh fruit ripening. In other words, NO has been demonstrated to delay fruit ripening generally, independently on whether fruits are climacteric or nonclimacteric (Palma et al., 2019). Integrating the results that the *LbNR* transcript amount is significantly positively correlated with the NO release during fruit ripening and that the VIGS-mediated *LbNR* gene silencing significantly decreased the NO production in ripe Lycium fruits, we could conclude that NR was partially responsible for NO biosynthesis in Lycium plants. To our knowledge, this is the first report that NR-derived NO formation has been involved in fruit maturation of medicinal plant (Manjunatha et al., 2010; Palma et al., 2019).

Coloration of fresh fruits is a natural and excellent phenotypic marker for studying the mechanisms behind the regulation of fruit ripening and pigment metabolism. Anthocyanins and PAs (also known as condensed tannins) are two common flavonoid compounds in fruits. The former is an important pigment contributing to the coloration of many fruits, including that of *L.* *ruthenicum* in this study (Li G. et al., 2019), while the latter as a colorless metabolite, is an essential taste factor affecting astringency and bitterness of fruits (Khoo et al., 2017). It has been well established that the biosynthesis of anthocyanins and PAs shares most steps in the flavonoid pathway, and leucoanthocyanidins are the first branch point between these two biosynthesis pathways (Jaakola, 2013; Xu et al., 2015). Leucoanthocyanidin reductase (LAR) and anthocyanidin reductase (ANR) function at the branching points of the PA pathway, leading to catechin and epicatechin synthesis, respectively (Jaakola, 2013; Xu et al., 2015). Given *LbNR*-derived NO declined anthocyanin accumulation and downregulated the structural gene expression in the anthocyanin-biosynthesis pathway but elevated PA production and upregulated the transcripts of *LbLAR* and *LbANR*, it is proposed that NO delays Lycium fruit coloration by inhibiting *de novo* anthocyanin biosynthesis. Integrating the finding that there is a significant negative relationship between the levels of anthocyanins and PAs during Lycium fruit ripening (**Figure S8**), it is shown that NO also redirects the flavonoid biosynthetic pathway from anthocyanin to PA production, thus indirectly regulating Lycium fruit coloration. Our results may establish a direct relationship between the NO signaling and the flavonoid biosynthetic pathway in fresh fruits.

Flavonoid biosynthetic pathway is transcriptionally controlled mainly by a ternary complex of MYB–bHLH–WD40 TFs (termed the MBW complex), in which R2R3-MYB TFs function mainly to determine the specificity of gene activation for anthocyanin and PA biosynthesis (Jaakola, 2013; Xu et al., 2015; Allan and Espley, 2018). So far, mutiple flavonoid-related MYB activators have been identified in a great deal of plant species, among which some co-regulates both anthocyanin and PA accumulation, but others specific to one of them (Nesi et al., 2001; Bogs et al., 2007; James et al., 2017; An et al., 2018; Li C. et al., 2019). Besides acting as positive regulators, MYB TFs also act as negative regulators (or repressors) in the regulation of flavonoid biosynthesis. In the present work, four R2R3-MYB type TFs, e.x. *LrAN2*, *LrMYB1, LrMYB3*, and *LrMYB44* were inactivated by NO, while two 1R-MYB type TFs, e.x. LrMYB30 and LrMYB73 were activated by NO, at transcriptional level. So far there is not a report for 1R-MYB type TFs involved in flavonoid metabolism (Ma and Constabel, 2019), but the present work demonstrated in Lycium plants that it is related with PA biosynthesis. Except MYB TFs, both the examined bHLH TFs and WD40 proteins that were positively correlated with anthocyanins were negatively correlated with PAs. It is shown in the transcriptional level that PA-related MBW activators negatively correlated with anthocyanin-related MBW activators in Lycium plants (data not shown). Whether there is a direct antagonism between those R2R3-MYB type TFs and those 1R-MYB type TFs in Lycium fruits needs to be further investigated in the future. Moreover, genome-wide characterization of the MBW complex that coordinated regulation of PA and anthocyanin accumulation, or specific to each of them, is also urgently needed for Lycium plants.

It is well known that the fruit ripening process is regulated by various phytohormone interactions, in which ethylene and ABA make a positive effect, while IAA, CTK, and GA exert a negative one (Gapper et al., 2013; Guo et al., 2018). In consideration of the fact that there is neither ethylene release nor respiration rate increase during Lycium fruit ripening (Feng and Zhang, 2010), it is proposed that Lycium fruit belongs to the nonclimacteric group. We have previously documented that ABA acts as a ripening enhancing hormone in Lycium fruits both by genetic and pharmacological approaches (Li G. et al., 2019). In the present work, we further demonstrated that NO attenuated the coloration of Lycium fruits by antagonizing the action of ABA, thereby delaying the fruit ripening. The interaction between NO signaling and ABA signaling has been shown to be involved in many plant physiological processes, e.x. seed germination/dormancy, stomatal movement, leaf senescence, fruit ripening, abiotic stress response, *etc*. (Zhang et al., 2019). Within these physiological processes, NO and ABA have either synergetic or antagonistic functions (Prakash et al., 2019). Inhibition of ABA biosynthesis, promotion of ABA decomposition, and inhibition of ABA signal transduction may be behind the mechanisms why NO antagonizes ABA (Prakash et al., 2019). Of course, this needs to be further validated in Lycium fruits. It has been established in stomatal cells that NR-derived NO, which was induced by ABA, was involved in ABA-mediated stomatal movement (Kolbert et al., 2019). On the contrary, in the present work, we found that ABA inhibited NO production in Lycium fruits though the mechanism remains unclear. Maybe it is the ABA-induced reactive oxygen species accumulation which directly reacted with NO, thus declining the endogenous NO level (Prakash et al., 2019). Taken together, our results may provide particularly comprehensive information about NO–ABA–anthocyanins interplay during Lycium fruit development and ripening. Nevertheless, little is known about the interplay between NO and other regulators involved in fruit ripening of Lycium plants.

Although we highlighted the NO-mediated transcriptional modification of flavonoid biosynthetic pathway, it should be kept in mind that NO exerts a signaling function also by posttranslational modification of target proteins, *e.g.*, protein S-nitrosylation and nitration (Feng et al., 2019). In *Arabidopsis*, the addition of either of the NO donors SNP or GSNO inhibited the DNA-binding capacity of the minimal DNA-binding domain of the TF R2R3-MYB2 due to the S-nitrosylation of Cys53 or the S-nitrosylation of AtMYB30 at Cys49 and Cys53 (Serpa et al., 2007; Tavares et al., 2014). It is speculated, therefore, that NO regulates R2R3-MYB activity at both the transcriptional level and the posttranslational level, thus inhibiting anthocyanin biosynthesis in Lycium fruits. However, the mechanism underlying the NO activation of PA-specific 1R-MYB TFs in these plants remains unclear. In the future, the NO-mediated posttranslational modification of the key enzymes involved in the biosynthesis of anthocyanins and PAs should also be addressed. Morover, as one of the metabolites of nitrate nutrients (Chamizo-Ampudia et al., 2017), the endogenous NO content can be fine-tuned by the regulation of NO_3_^−^ application. Therefore, Lycium fruit coloration/ripening can also be manipulated by the application of fertilizers that contain NO_3_^−^ (Jezek et al., 2018) as has been shown in *Arabidopsis*, grape berry and apple (Zhou et al., 2012; Habran et al., 2016; Wang et al., 2018). We look forward to this theme which will become a promising research project due to the versatile function of NO in regulating fresh fruit ripening, quality and shelf life.

## Data Availability Statement

The datasets generated for this study are available on request to the corresponding authors.

## Author Contributions

ZM, YC, and JZ designed the research. GL, BQ, and SL performed the experiments. GL and SL conducted the data analyses. YY, JZ, and WA conducted the field management work. ZM and GL wrote the manuscript. All authors contributed to the article and approved the submitted version.

## Funding

This work was jointly supported by the National High-tech R&D Program (863 Program, 2013AA102606-04), the National Natural Science Foundation of China (31660220), the Project of Agricultural Breeding of New Wolfberry Varieties in Ningxia (2013NYYZ0101), and the Self-option and Foundation of Ningxia Academy of Agriculture and Forestry Sciences (NKYJ-18-16).

## Conflict of Interest

The authors declare that the research was conducted in the absence of any commercial or financial relationships that could be construed as a potential conflict of interest.

## Supplementary Material

Table S1 Primers utilized in LbNR full length cioning.

Table S2 Primers utilized in qRT-PCR.

Figure S1 Transcripts changes of PA biosynthesis-related genes during the process of YF (upper column) and BF (down column) ripening, respectively. The error bars represent the SDs of three independent replicates. Different letters on the bars for the same species indicate significant differences between the treatments (p < 0.05).

Figure S2 Effect of *LbNR* silencing on PA biosynthesis-related gene expression, both for YF and BF. The error bars represent the SDs of three independent replicates. The asterisks on the bars for the same species indicate significant differences between the treatments (p < 0.01).

Figure S3 Effect of exogenous SNP on PA biosynthesis-related gene expression, both for YF and BF. The error bars represent the SDs of three independent replicates. The asterisks on the bars for the same species indicate significant differences between the treatments. “*” indicates p < 0.05, and “**” indicates p < 0.01.

Figure S4 The relationship between the release of NO and the accumulation of ABA during the process of YF **(A)** and BF **(B)** ripening, respetively. p < 0.01.

Figure S5
*LbNCED1* silencing enhanced PA accumulation, both for YF **(A)** and BF **(B)**. The error bars represent the SDs of three independent replicates. The asterisks on the bars for the same species indicate significant differences between the treatments. “*” indicates p < 0.05, and “**” indicates p < 0.01.

Figure S6 Transcriptional response of LrMYB TFs to *LbNR* silencing in BF. The error bars represent the SDs of three independent replicates. The asterisks on the bars for the same species indicate significant differences between the treatments. “*” indicates p < 0.05, and “**” indicates p < 0.01.

Figure S7 Transcriptional response of LrMYB TFs to *LbNCED1* silencing in BF. The error bars represent the SDs of three independent replicates. The asterisks on the bars for the same species indicate significant differences between the treatments. “*” indicates p < 0.05, and “**” indicates p < 0.01.

Figure S8 The relationship between anthocyanins and PAs during the process of YF **(A)** and BF **(B)** ripening, respetively. p < 0.01.
