# Supplementary material for: LbNR-Derived Nitric Oxide Delays Lycium Fruit Coloration by Transcriptionally Modifying Flavonoid Biosynthetic Pathway
Source: Front Plant Sci. 2020 Aug 13;11:1215. doi: 10.3389/fpls.2020.01215 (PMC7438876; doi:10.3389/fpls.2020.01215)
Supplement: Table S1 — Primers utilized in LbNR full length cioning. [file Table_1.docx]

**Table S1. Primers used in *LbNR* full length cloning**

| Primer name | Primer sequence（5’-3’） | Primer length (bp) |
| --- | --- | --- |
| oLigd(T) | GCTGTCAACGATACGCTACGTAACGGCATGACAGTGTTTTTTTTTTTTTTTTTT | 54 |
| *LbNR*-Segment-F | CCATGGCTGCATCTGTTGAAAATCG | 25 |
| *LbNR*-Segment –R  *LbNR*-Full length-F  *LbNR*-Full length-R | CATGACATTCCAAATAAGGTTCTCG  ATGGCTGCATCTGTTGAAAAT  AATTTATTACTGCAGATTGTTGTTA | 25  21  25 |
| 3 outer | GCTGTCAACGATACGCTACGTAACG | 25 |
| 3 inner | CGCTACGTAACGGCATGACAGTG | 23 |
| 5 outer | GGC CAC GCG TCG ACT AGT ACG GGI IGG GII GGG IIG | 36 |
| 5 inner | GGCCAC GCG TCG ACT AGT AC | 20 |
| *LbNR* 3’GSPⅠ | TGGAAGAATGGTGAAATGGTTGAAG | 25 |
| *LbNR* 3’GSPⅡ | CCCGAGAACCTTATTTGGAATGTCA | 25 |
| *LbNR* 5’GSPⅠ | ACAAGCGTAACTGGCAACTCCCTGG | 25 |
| *LbNR* 5’GSPⅡ | GACCAGACCAGTAACTTCCACGGAC | 25 |
| Adaptor5’  Adaptor3’ | CGACGACAAGACCCT  GAGGAGAAGAGCCCT | 15  15 |
