# Supplementary material for: LbNR-Derived Nitric Oxide Delays Lycium Fruit Coloration by Transcriptionally Modifying Flavonoid Biosynthetic Pathway
Source: Front Plant Sci. 2020 Aug 13;11:1215. doi: 10.3389/fpls.2020.01215 (PMC7438876; doi:10.3389/fpls.2020.01215)
Supplement: Table S2 — Primers utilized in qRT-PCR. [file Table_2.docx]

**Table S2. Primers in quantitative PCR**

| Primer name | Primer sequence (5’-3’) | | | Primer length |
| --- | --- | --- | --- | --- |
| *LbActin* F | GGAAACATAGTGCTCAGTGGTG | | | 22 |
| *LbActin* R | GCTGAGGGAAGCCAAGATAG | | | 20 |
| *LbNCED1* F | GTTTCCGCTGTAACTGTCTCAAG | | | 23 |
| *LbNCED1* R | CATAGAAGTAAAGACCTCACCACCT | | | 25 |
| *LbNR* F | GCAGCAGCAATGGCTTTAGA | | | 23 |
| *LbNR* R | GGTGACCGGAAGTGATTGAGT | | | 25 |
| *LrANS* F | GATCCACCTCGATTCCCACC | | | 21 |
| *LrANS* R | TGTTCATCCTTTTTGGCGGC | | | 22 |
| *LrCHS1* F ^d^ | AAATGAGAAAGGCCTCTGCA | | | 20 |
| *LrCHS1* R ^d^ | GACCCACAGTAGAAGACCAACC | 22 | | |
| *LrCHI2* F ^d^ | TGCAAAGATTTCATCCACCA | | 20 | |
| *LrCHI2* R ^d^ | AAATGCAAAAGCCAACATGA | | 20 | |
| *LrF3H* F ^d^ | GAGCTCGCTAGGCTCAAGAA | | 20 | |
| *LrF3H* R ^d^ | CACGAGTAGCATCTTTTTCAACA | | 23 | |
| *LrF3’H* F ^d^ | AACATGGAGGAAGCATTTGG | | 20 | |
| *LrF3’H* R ^d^ | TGGCCAGATGTGTTAAAAGC | | 20 | |
| *LrF3’5’H* F ^d^ | TGGGAACTTTGGTTCATTCA | | 20 | |
| *LrF3’5’H* R ^d^ | TAGCCAAATCGACTCCCAAA | | 20 | |
| *LrUF3GT* F ^d^ | GGCAAAATGTTAAAAGGCTAAAA | | 23 | |
| *LrUF3GT* R ^d^ | GACTGAAGCAAATTCCAGCTAA | | 22 | |
| *LrDFR2* F ^d^ | GGCCTTGAGGAAATGTTTGA | | 20 | |
| *LrDFR2* R ^d^ | TCTATGCGCGTACATGGAAA | | 20 | |
| *LrAN2* F ^d^ | AGCTTCATCCATCAGTAGCATT | | 22 | |
| *LrAN2* R^d^ | CCCCGATGGTGTAAATCC | | 18 | |
| *LrJAF13* F ^d^ | AATCCAAGTCTACGGGATTGAA | | 22 | |
| *LrJAF13* R ^d^ | CCTCCGAGGACATGATTGAC | | 20 | |
| *LrAN1b* F ^d^ | GAAAGCGAGCATTCTGGAAG | | 20 | |
| *LrAN1b* R ^d^ | ATCACACACATCGTCGGTTG | | 20 | |
| *LrAN11* F ^d^ | CTGCGACACGTGATTGGAT | | 19 | |
| *LrAN11* R ^d^ | CCAAGCTTTAACCCCTTTCC | | 20 | |
| *LbANR* F ^a^ | TGGGCACGACAAACATCCTC | | 20 | |
| *LbANR* R ^a^ | CAGTGTTGCTCTTGCCATGT | | 20 | |
| *LbLAR* F ^a^ | ATTCAGTGGCTTCTTGGCCTT | | 21 | |
| *LbLAR* R ^a^ | AGCACCAGCCACAAAATAAGC | | 21 | |
| *LrMYB30* F ^c^ | CTGCTGCCTTCCCTACTACA | | 20 | |
| *LrMYB30* R ^c^ | GAGGTTCTTCA TCTTTCGGTC | | 21 | |
| *LrTTG1-like* F ^b^ | GATCATCGCTTGCTGATGCG | | 20 | |
| *LrTTG1-like* R ^b^ | CATTGCCAGTGGGAGTGAGA | | 20 | |

**REFERENCES**

^a^Chen, C., Xu, M., Wang, C., Qiao, G., Wang, W., Tan, Z., et al. (2017). [Characterization of the *Lycium barbarum* fruit transcriptome and development of EST-SSR markers.](https://www.ncbi.nlm.nih.gov/pubmed/29125846) *PLoS One* 12, e0187738. doi: 10.1371/journal.pone.0187738

^b^Yan, L., Chen, J.W., Wang, C.P., Tong, Q., Wang, C., Qiao, G.X., et al. (2019). Analysis of WD40 protein family based on transcriptome sequencing in *Lycium ruthenicum* Murr. *J. Nuclear Agricul. Sci*. 33, 482–489. doi: 10. 11869/j.issn.100-8551 (in Chinese with English abstract).

^c^Yan, L., Wang, C.P., Chen, J.W., Qiao, G.X., and Li, J. (2017). Analysis of MYB transcription factor family based on transcriptome sequencing in *Lycium ruthenicum* Murr. *Sci. Agricul. Sinica*. 50, 3991–4002. doi: 10.3864/j.issn.0578-1752.2017.20.013 (in Chinese with English abstract)

^d^Zeng, S., Wu, M., Zou, C., Liu, X., Shen, X., Hayward, A., et al. (2014). [Comparative analysis of anthocyanin biosynthesis during fruit development in two *Lycium* species.](https://www.ncbi.nlm.nih.gov/pubmed/24661321) *Physiol. Plant*. 150, 505–516. doi: 10.1111/ppl.12131
