# Supplementary figures and images for: LbNR-Derived Nitric Oxide Delays Lycium Fruit Coloration by Transcriptionally Modifying Flavonoid Biosynthetic Pathway
Source: Front Plant Sci. 2020 Aug 13;11:1215. doi: 10.3389/fpls.2020.01215 (PMC7438876; doi:10.3389/fpls.2020.01215)

Relative expression level

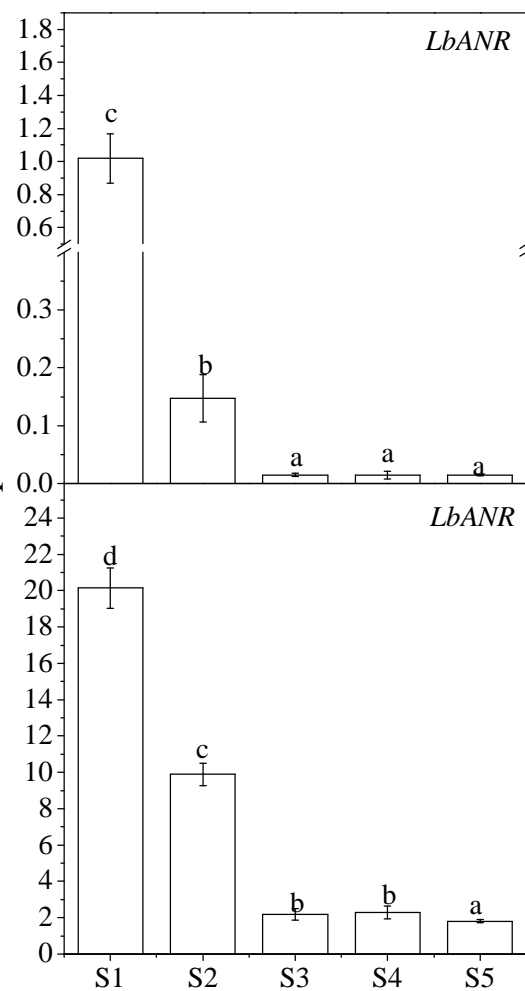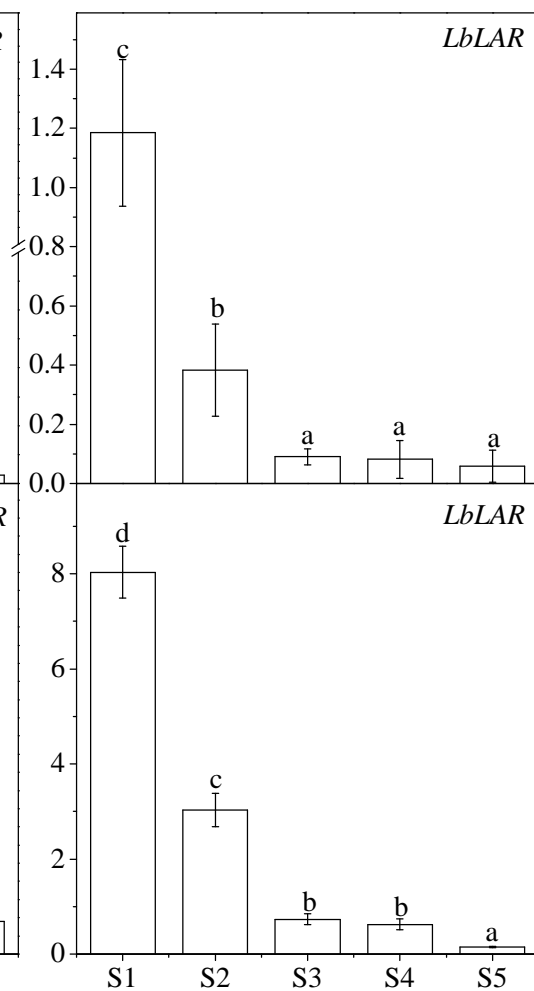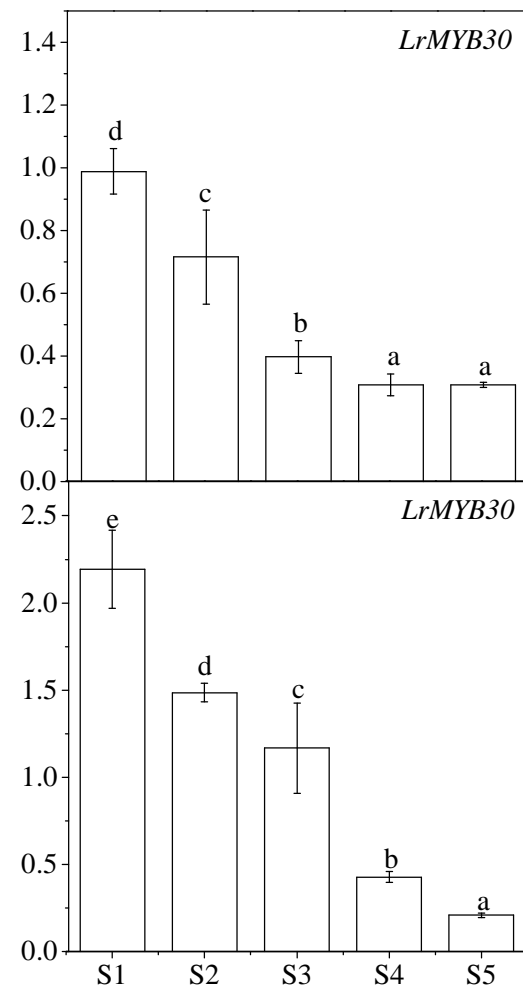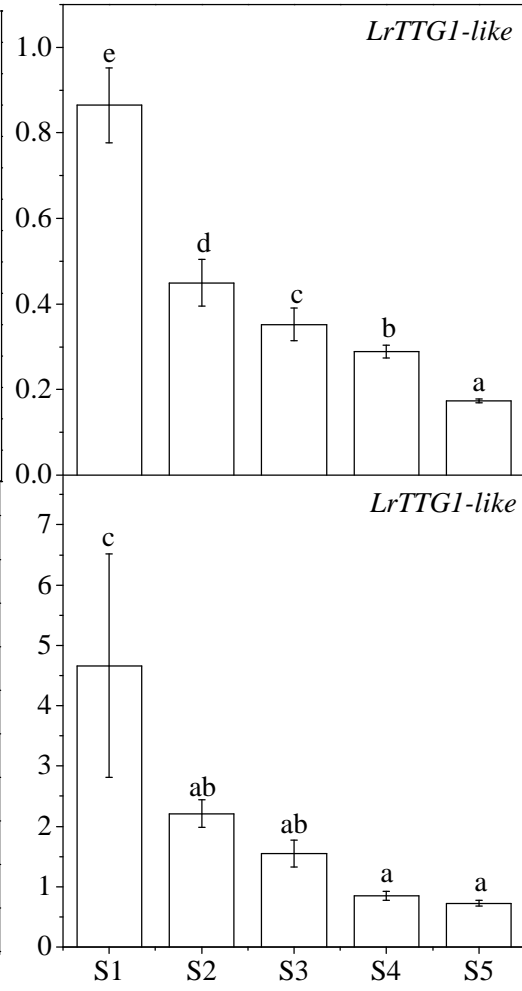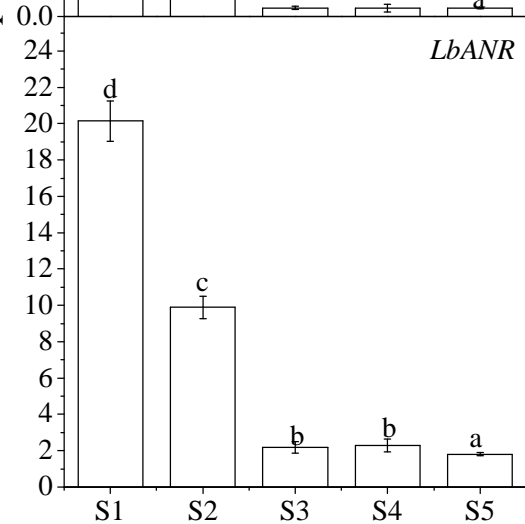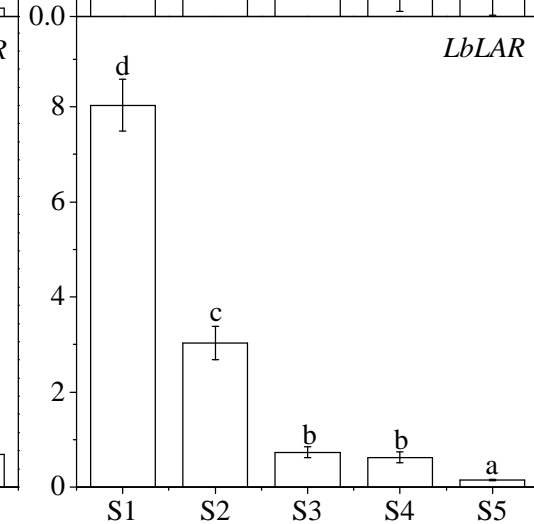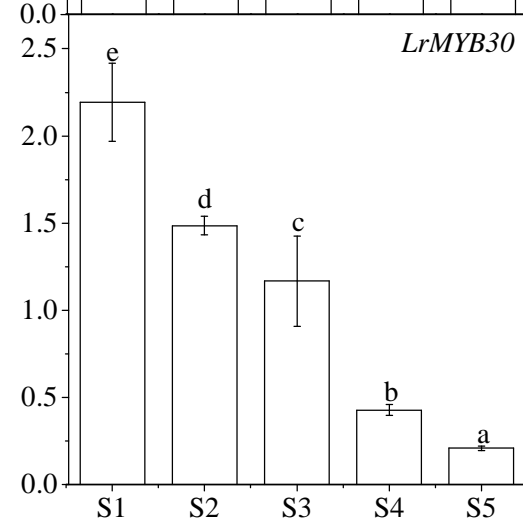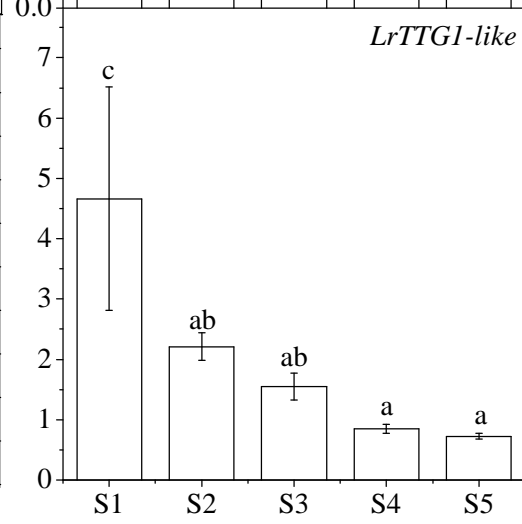

Supplement: Figure S1 — Transcripts changes of PA biosynthesis-related genes during the process of YF (upper column) and BF (down column) ripening, respectively. The error bars represent the SDs of three independent replicates. Different letters on the bars for the same species indicate significant differences between the treatments (p < 0.05). [file Image_1.pdf]

Relative expression level

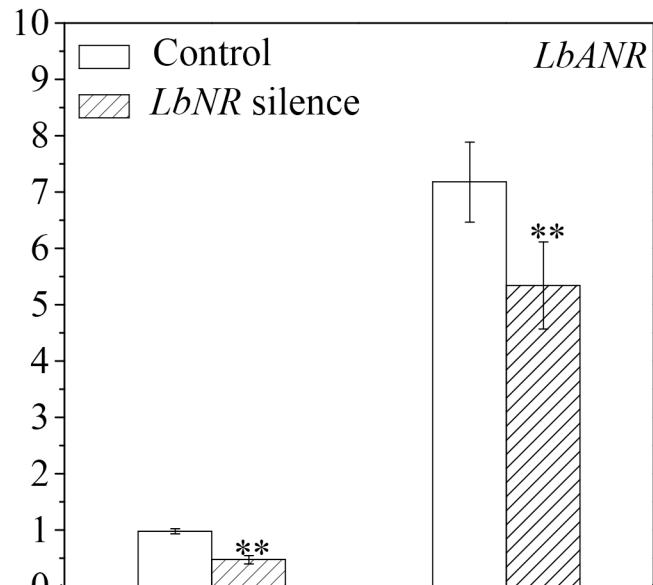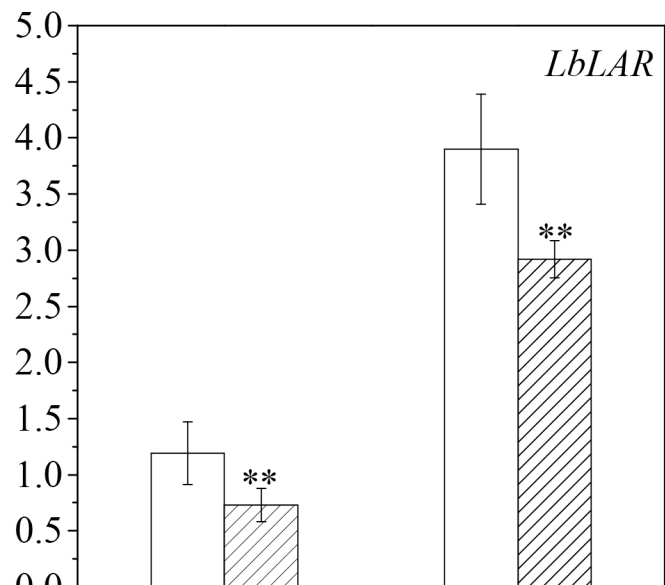

Relative expression level

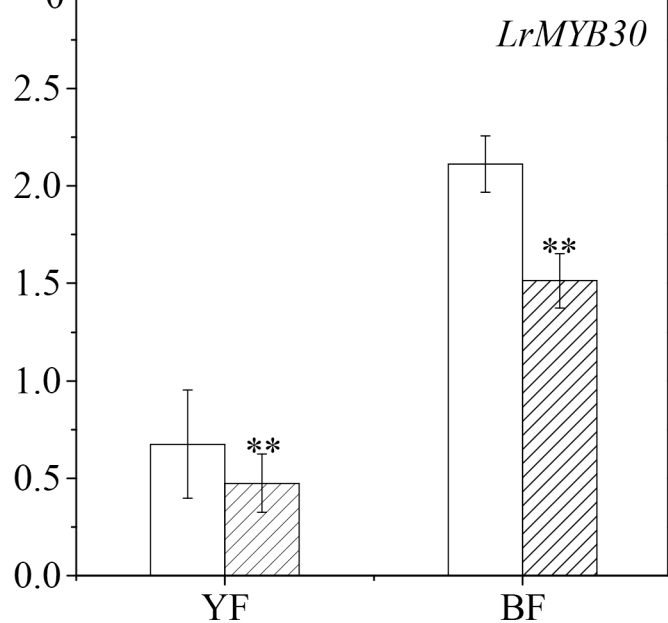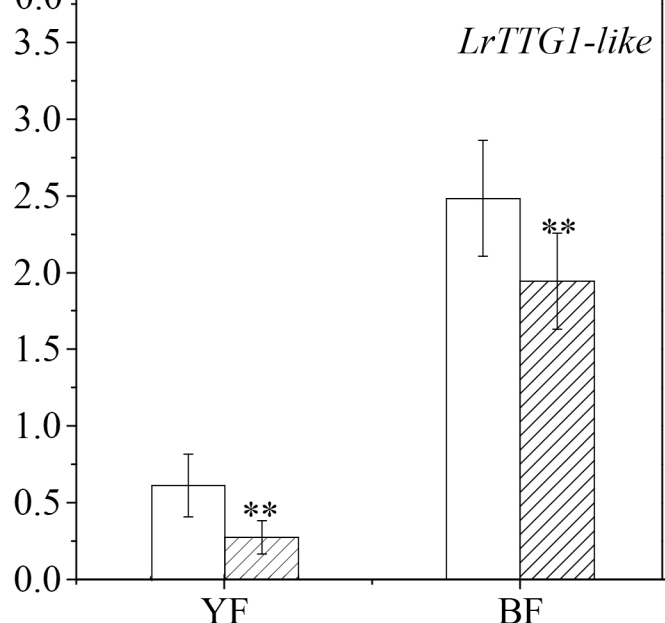

Supplement: Figure S2 — Effect of LbNR silencing on PA biosynthesis-related gene expression, both for YF and BF. The error bars represent the SDs of three independent replicates. The asterisks on the bars for the same species indicate significant differences between the treatments (p < 0.01). [file Image_2.pdf]

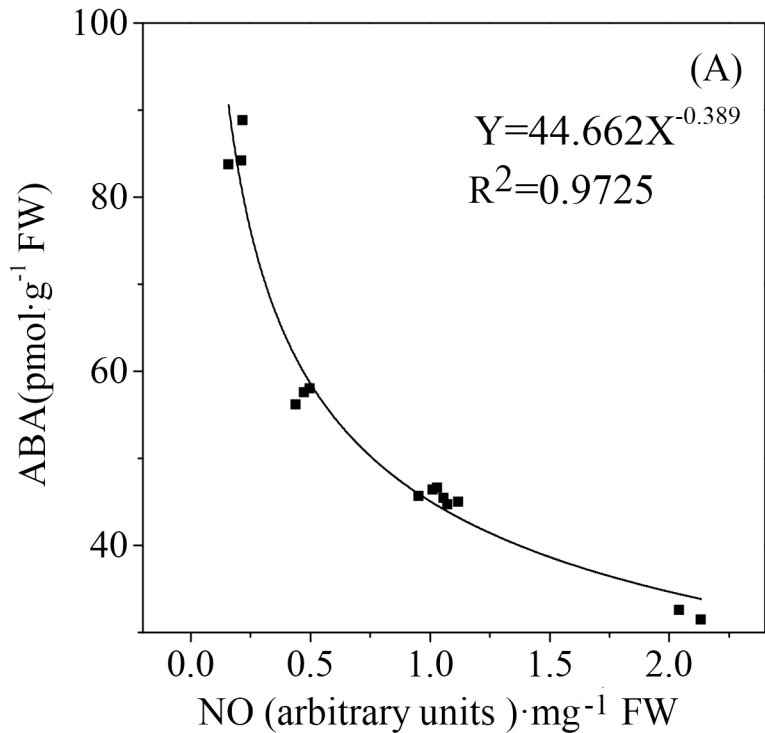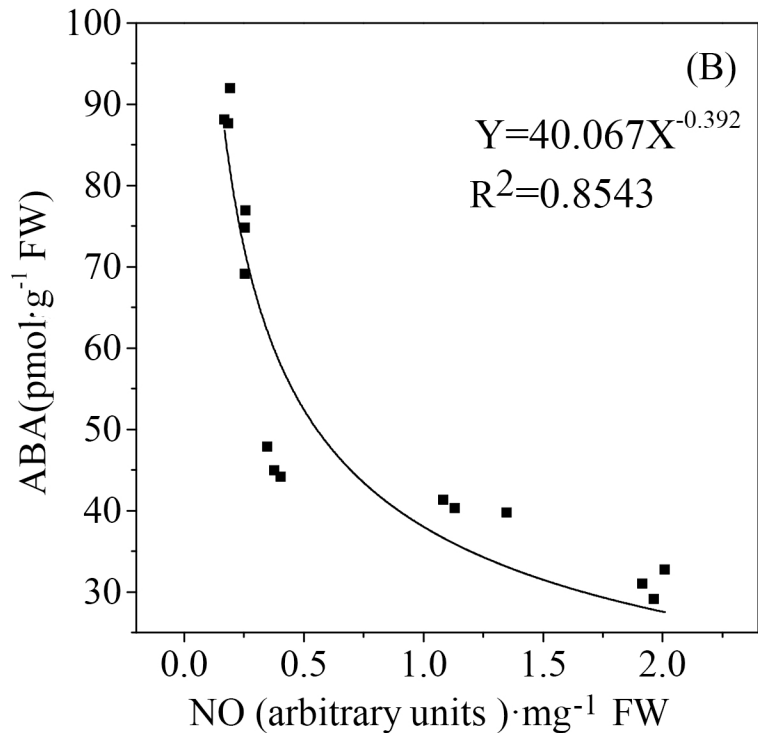

Supplement: Figure S4 — The relationship between the release of NO and the accumulation of ABA during the process of YF (A) and BF (B) ripening, respetively. p < 0.01. [file Image_4.pdf]

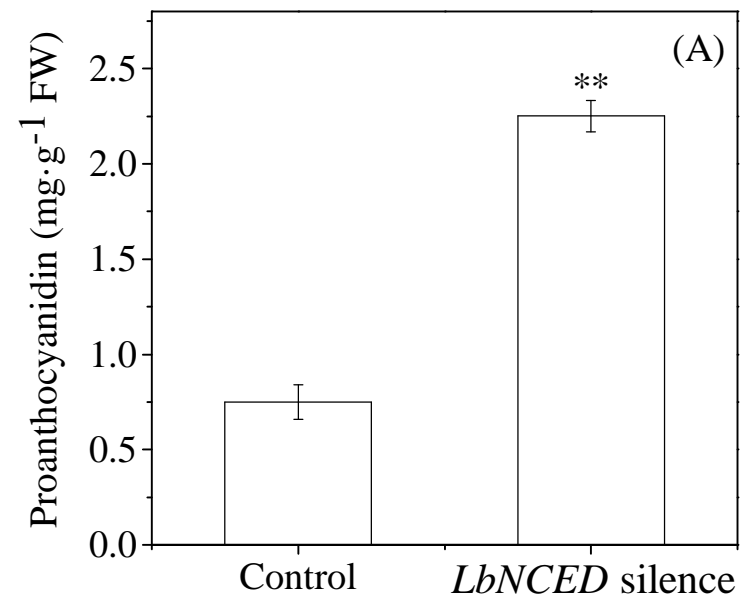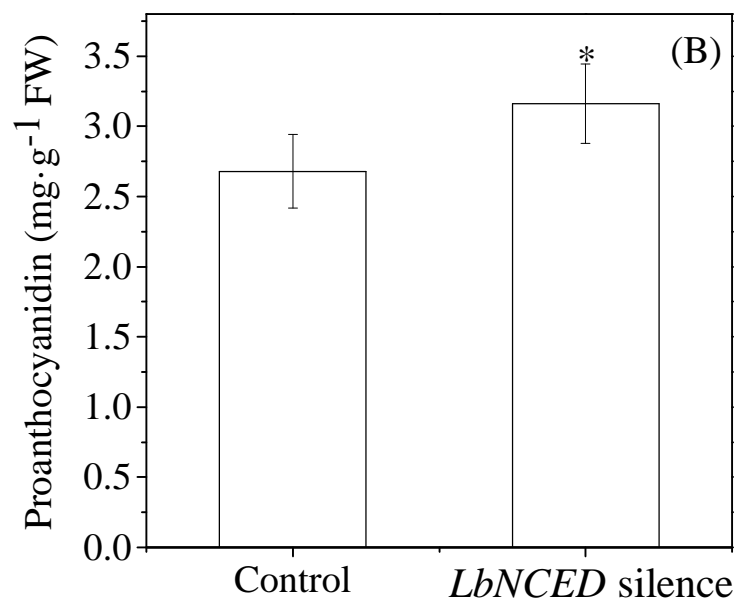

Supplement: Figure S5 — LbNCED1 silencing enhanced PA accumulation, both for YF (A) and BF (B). The error bars represent the SDs of three independent replicates. The asterisks on the bars for the same species indicate significant differences between the treatments. “*” indicates p < 0.05, and “**” indicates p < 0.01. [file Image_5.pdf]

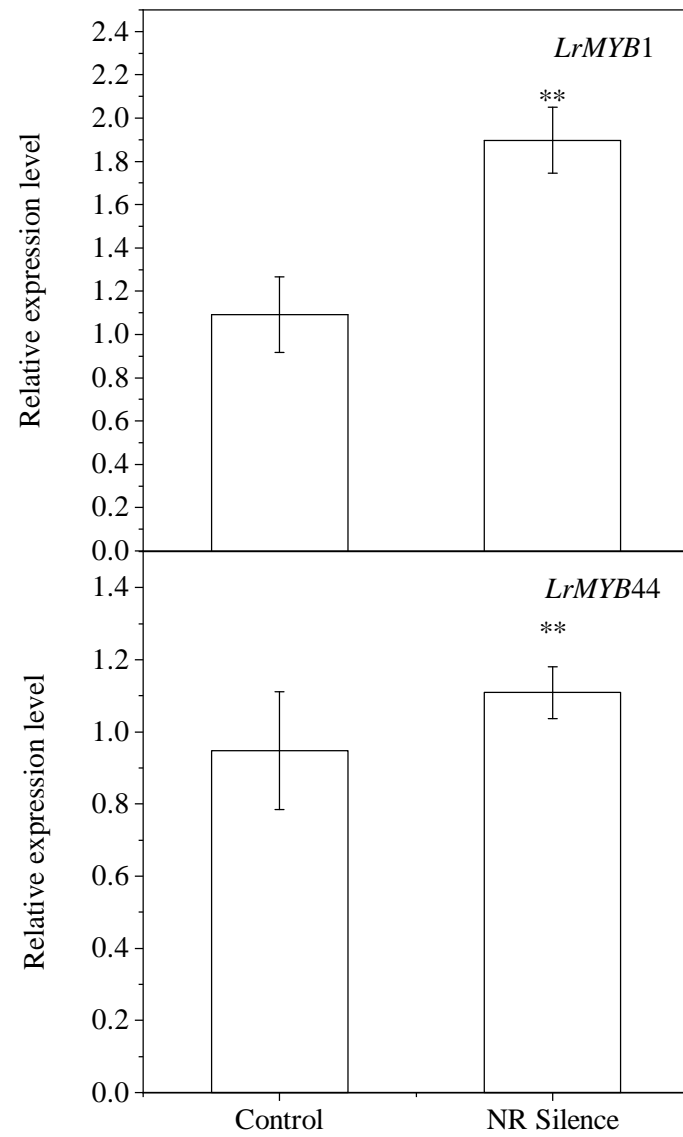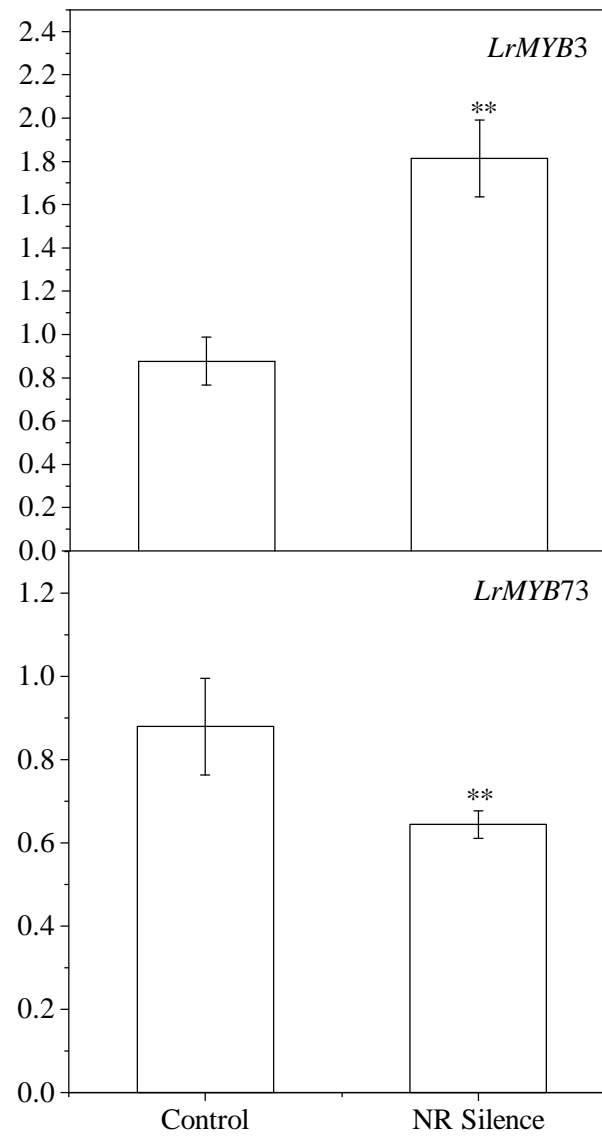

Supplement: Figure S6 — Transcriptional response of LrMYB TFs to LbNR silencing in BF. The error bars represent the SDs of three independent replicates. The asterisks on the bars for the same species indicate significant differences between the treatments. “*” indicates p < 0.05, and “**” indicates p < 0.01. [file Image_6.pdf]

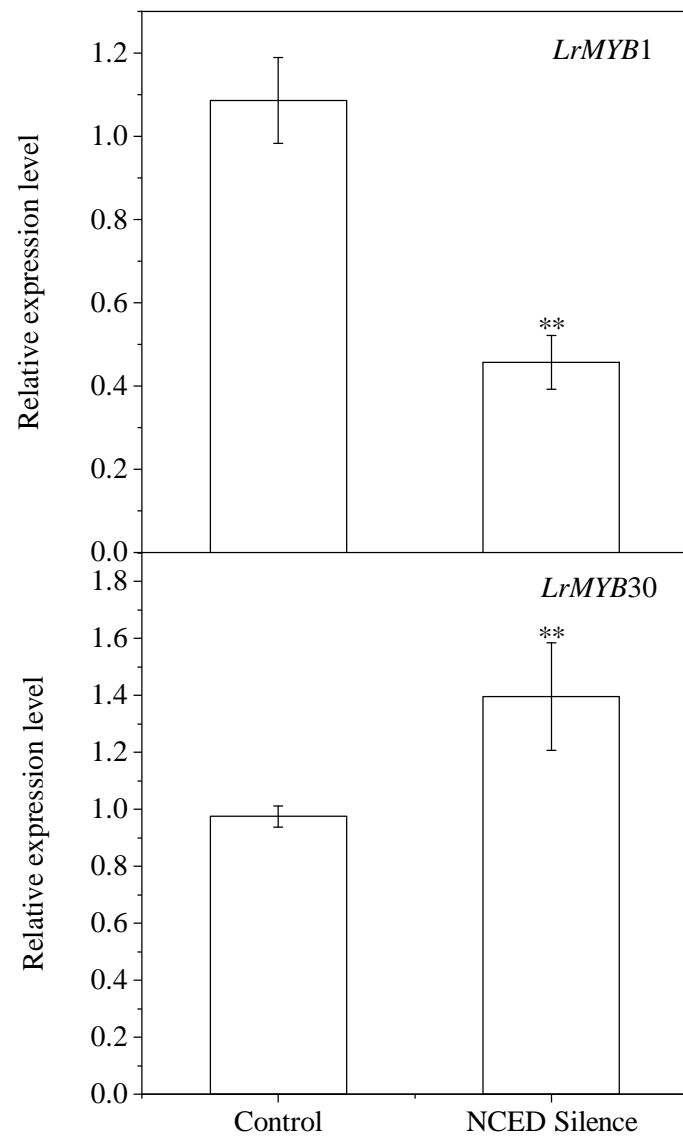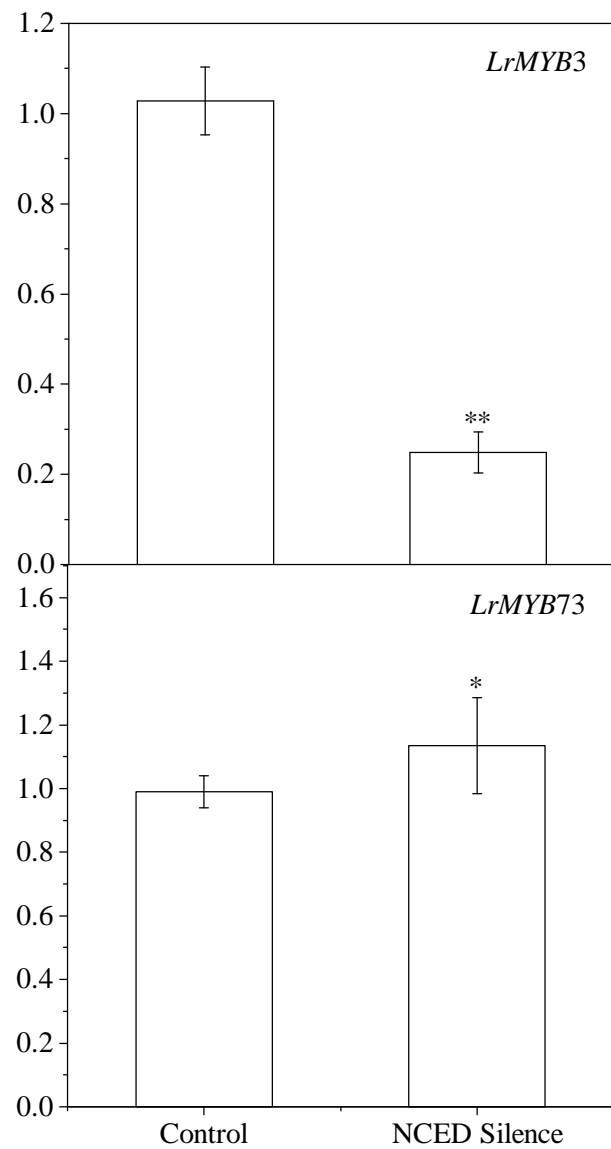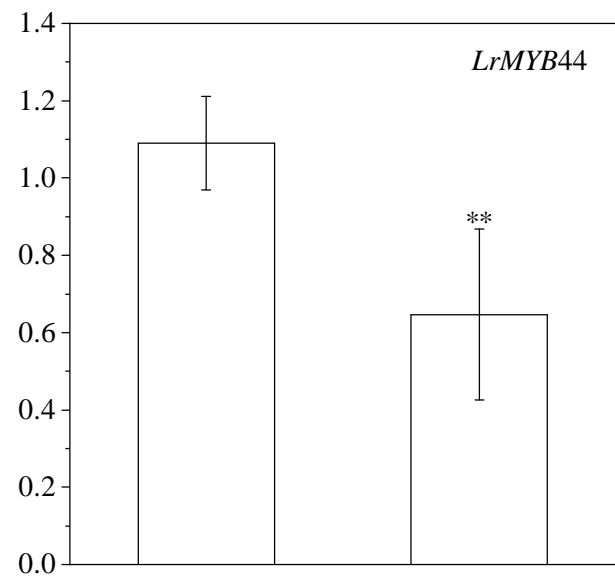

Supplement: Figure S7 — Transcriptional response of LrMYB TFs to LbNCED1 silencing in BF. The error bars represent the SDs of three independent replicates. The asterisks on the bars for the same species indicate significant differences between the treatments. “*” indicates p < 0.05, and “**” indicates p < 0.01. [file Image_7.pdf]

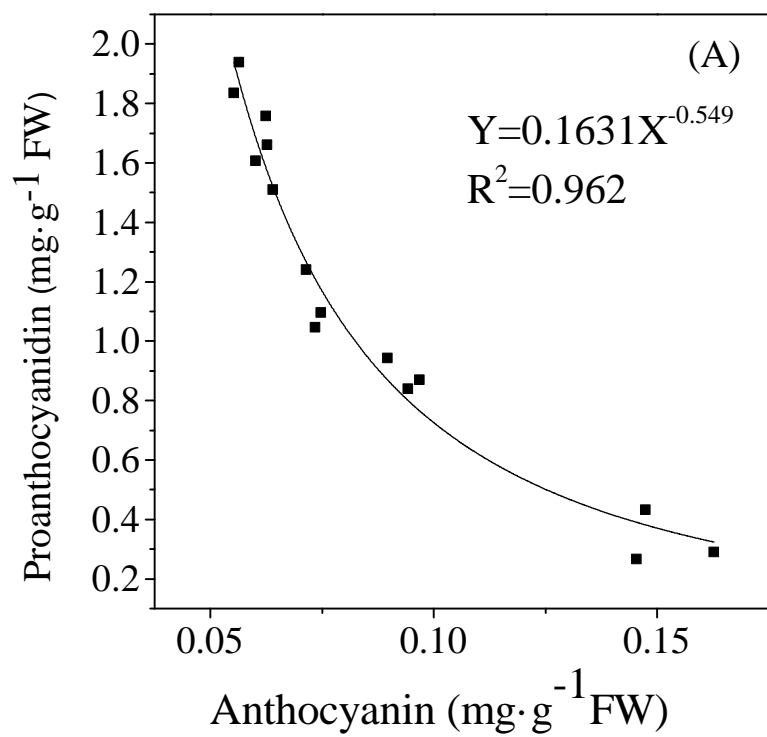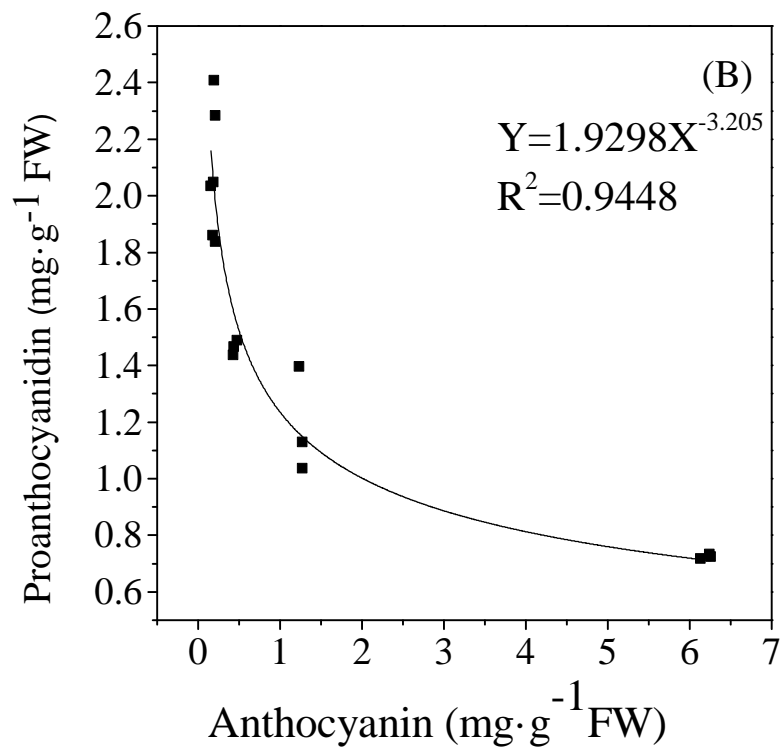

Supplement: Figure S8 — The relationship between anthocyanins and PAs during the process of YF (A) and BF (B) ripening, respetively. p < 0.01. [file Image_8.pdf]
