# Supplementary material for: LbNR-Derived Nitric Oxide Delays Lycium Fruit Coloration by Transcriptionally Modifying Flavonoid Biosynthetic Pathway
Source: Front Plant Sci. 2020 Aug 13;11:1215. doi: 10.3389/fpls.2020.01215 (PMC7438876; doi:10.3389/fpls.2020.01215)
Supplement: Figure S3 — Effect of exogenous SNP on PA biosynthesis-related gene expression, both for YF and BF. The error bars represent the SDs of three independent replicates. The asterisks on the bars for the same species indicate significant differences between the treatments. “*” indicates p < 0.05, and “**” indicates p < 0.01. [file Image_3.pdf]

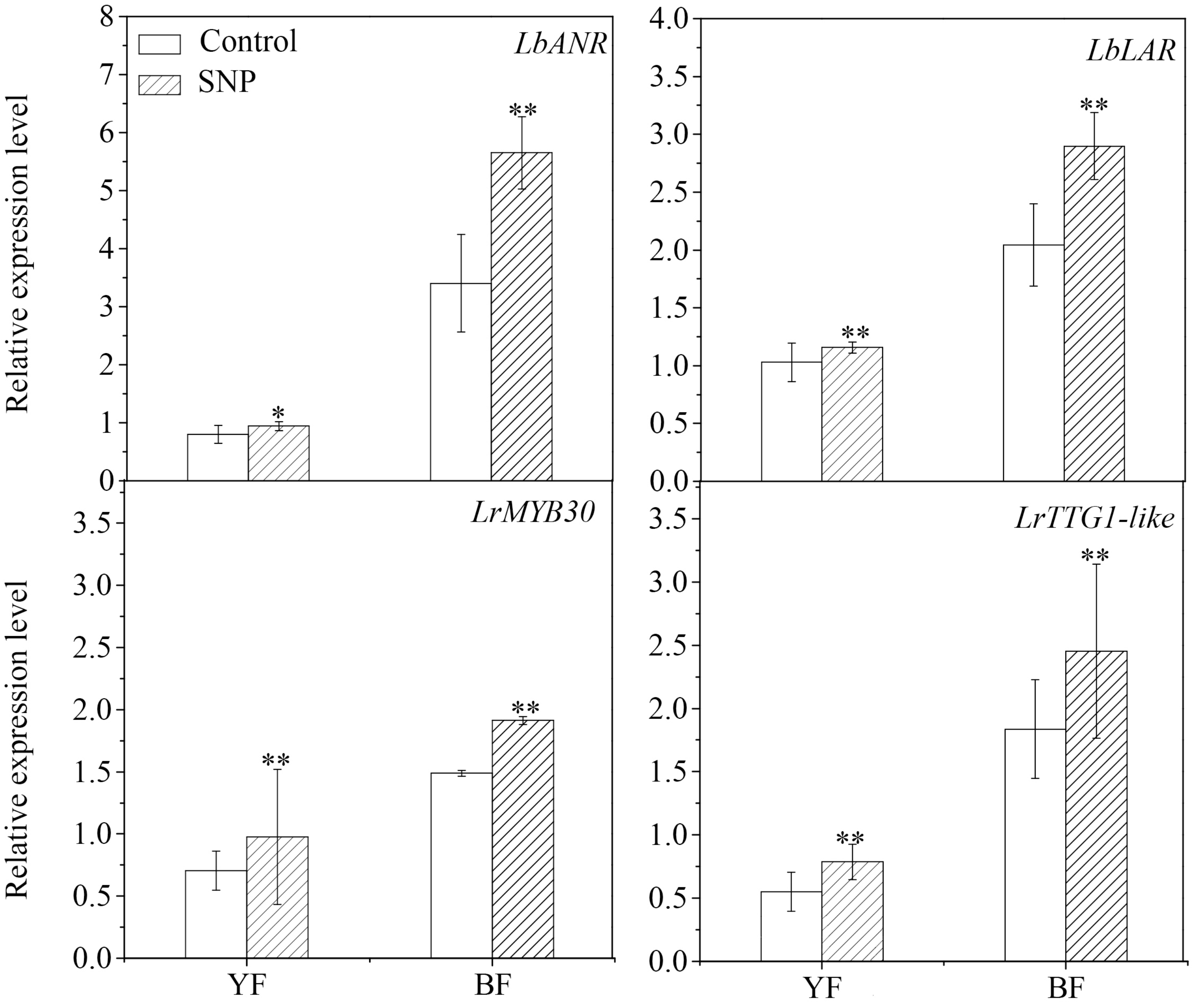

Control
SNP
LbANR
LbLAR
LrMYB30
LrTTG1-like
Relative expression level
Relative expression level
YF
BF
YF
BF
